# Transcriptome Analyses of Inhibitor-treated Schistosome Females Provide Evidence for Cooperating Src-kinase and TGFβ Receptor Pathways Controlling Mitosis and Eggshell Formation

**DOI:** 10.1371/journal.ppat.1003448

**Published:** 2013-06-13

**Authors:** Christin Buro, Katia C. Oliveira, Zhigang Lu, Silke Leutner, Svenja Beckmann, Colette Dissous, Katia Cailliau, Sergio Verjovski-Almeida, Christoph G. Grevelding

**Affiliations:** 1 Institute of Parasitology, Justus-Liebig-University Giessen, Giessen, Germany; 2 Departamento de Bioquímica, Instituto de Química, Universidade de São Paulo, São Paulo, Brasil; 3 CIIL - Center of Infection and Immunity of Lille, Université Lille Nord de France, Inserm U1019, CNRS-UMR 8204, Institut Pasteur de Lille, Lille, France; 4 Laboratoire de Régulation des Signaux de Division, Université Lille 1 Sciences et Technology, EA 4479, IFR 147, Villeneuve d'Ascq, France; Rush University Medical Center, United States of America

## Abstract

Schistosome parasites cause schistosomiasis, one of the most prevalent parasitemias worldwide affecting humans and animals. Constant pairing of schistosomes is essential for female sexual maturation and egg production, which causes pathogenesis. Female maturation involves signaling pathways controlling mitosis and differentiation within the gonads. *In vitro* studies had shown before that a Src-specific inhibitor, Herbimycin A (Herb A), and a TGFβ receptor (TβR) inhibitor (TRIKI) have physiological effects such as suppressed mitoses and egg production in paired females. As one Herb A target, the gonad-specifically expressed Src kinase SmTK3 was identified. Here, we comparatively analyzed the transcriptome profiles of Herb A- and TRIKI-treated females identifying transcriptional targets of Src-kinase and TβRI pathways. After demonstrating that TRIKI inhibits the schistosome TGFβreceptor SmTβRI by kinase assays in *Xenopus* oocytes, couples were treated with Herb A, TRIKI, or both inhibitors simultaneously *in vitro*. RNA was isolated from females for microarray hybridizations and transcription analyses. The obtained data were evaluated by Gene Ontology (GO) and Ingenuity Pathway Analysis (IPA), but also by manual classification and intersection analyses. Finally, extensive qPCR experiments were done to verify differential transcription of candidate genes under inhibitor influence but also to functionally reinforce specific physiological effects. A number of genes found to be differentially regulated are associated with mitosis and differentiation. Among these were calcium-associated genes and eggshell-forming genes. *In situ* hybridization confirmed transcription of genes coding for the calcium sensor hippocalcin, the calcium transporter ORAI-1, and the calcium-binding protein calmodulin-4 in the reproductive system pointing to a role of calcium in parasite reproduction. Functional qPCR results confirmed an inhibitor-influenced, varying dependence of the transcriptional activities of Smp14, Smp48, fs800, a predicted eggshell precursor protein and SmTYR1. The results show that eggshell-formation is regulated by at least two pathways cooperatively operating in a balanced manner to control egg production.

## Author Summary

As one of the most prevalent parasitic infections worldwide, schistosomiasis is caused by blood-flukes of the genus *Schistosoma*. Pathology coincides with egg production, which is started upon pairing of the dioeciously living adults. A constant pairing contact is required to induce mitoses and differentiation processes in the female leading to the development of the gonads. Although long known, the molecular processes controlling gonad development or egg-production in schistosomes or other platyhelminths are largely unknown. Using an established *in vitro*-culture system and specific, chemical inhibitors we have obtained first evidence in previous studies for the participation of signal transduction processes playing essential roles in controlling mitoses, differentiation and egg production. In the present study we applied combinatory inhibitor treatments combined with subsequent microarray and qPCR analyses and demonstrate for the first time that cooperating Src-Kinase- und TGFβ-signaling pathways control mitoses and egg formation processes. Besides direct evidence for managing transcription of eggshell-forming genes, new target molecules of these pathways were identified. Among these are calcium-associated genes providing a first hint towards a role of this ion for reproduction. Our finding shed first light on the signaling mechanisms controlling egg formation, which is important for life-cycling and pathology.

## Introduction

Blood-dwelling endoparasites of the genus *Schistosoma* are the only trematodes that have evolved a gender dimorphism [1], [2]. These parasites cause schistosomiasis, which is of worldwide significance for humans and animals in tropical and sub-tropical areas [3]. About 780 million people live in endemic areas being at risk of schistosomiasis, of which 200 million are infected generating annual losses of 1.7 to 4.5 million disability adjusted life years (DALYs) of humans as determined by the Global Burden of Disease Programme [4], [5]. Living in the abdominal veins of their vertebrate hosts, adult paired females produce up to 300 eggs per day. Half of these eggs penetrates the epithelia and reach the gut lumen (e.g. *S. mansoni*) or the bladder (*S. haematobium*) to be transported to the environment for continuing the life cycle. The remaining eggs migrate via the blood stream to different organs such as spleen and liver, where they get trapped causing granuloma formation and liver cirrhosis [6], [7].

A unique biological feature of schistosomes is the dependency of the sexual maturation of the female on a constant pairing contact with the male. Following pairing, mitoses and differentiation are induced in the female leading to the differentiation of the reproductive organs, ovary and vitellarium [8], [9]. Regarding the importance of eggs for continuing the life cycle and provoking pathogenesis, a number of studies focused on the identification and characterization of genes controlling reproductive development of this parasite [8], [10]–[14]. Furthermore, genome and transcriptome projects have unravelled the parasite's genetic repertoire [15]–[17]. Several members of the TGFβ signaling pathway were identified, which is a highly conserved pathway throughout the animal kingdom. Among these, the type I TGFβ receptor (SmTβRI; Smp_049760; [18]), a type IIb activin receptor called SmRK2 (SmActRIIb; Smp_144390; [19]), SmSmad4 (Smp_033950; [20]), SmSmad2 (Smp_085910; [21]), and SmFKBP12 (Smp_079230; [22]) were identified. Localisation studies revealed a preferential expression of the listed molecules within the reproductive organs. SmTβRI transcripts were localised within the vitellarium and ovary as well as the parenchyma of both genders [23]. Furthermore, expression of SmTβRI protein was also found at the surface of male parasites [18]. SmActRIIb was found to be expressed in males and females, and localised on the tegumental surface of the gynaecophoral canal and some parenchymatic cells [19]. SmSmad4 was detected within epithelia surrounding the gut and vitellarium, as well as the subtegument and muscles of males [20] and SmSmad2 within the vitellarium, the developing egg, and the ovary of the female worm, but also in the testes and tubercles of the male [24]. SmFKBP12 co-localised with SmTβRI in the female gonads as well as the parenchyma of the adult schistosomes [23].

In the animal kingdom, the TGFβ pathway controls proliferation and differentiation processes [25]. First studies to elucidate the functional meaning of the schistosome TGFβ pathway have included ligand-induction and inhibitor-suppression approaches [11], [26]. Using human TGFβ (hTGFβ) to induce the TGFβ pathway in adults *in vitro*, a recent study showed that genes related to morphology, development, and cell cycle were differentially transcribed [27]. Earlier studies, based on the use of a specific TβRI kinase inhibitor (TRIKI) in adult schistosomes *in vitro* to suppress the TGFβ pathway, provided first evidence for its role in regulating mitotic activity and egg production in paired *S. mansoni* females [11]. Using a similar inhibitor approach with adults *in vitro* indicated the additional influence of (a) Src kinase-containing pathway(s) on these processes in paired *S. mansoni* females. Based on the discovery of the gonad-specific expression of the cellular Src tyrosine kinase SmTK3 (Smp_151300; [28]), inhibition experiments with the Src-kinase inhibitor Herbimycin A (Herb A) led to reduced mitotic activity and egg production in paired females as well [29]. The comparison of both inhibitor treatments pointed to a stronger reduction of both parameters following Herb A treatment [11]. The strongest influence on the mitotic activity and egg production was observed by combining both inhibitors.

In this study, we investigated the inhibitory impact of TRIKI, Herb A, or the combined compounds on the transcriptome of female schistosomes using a microarray approach and comprehensive qPCR analyses. Besides the identification of a large number of genes, which were differentially transcribed upon inhibitor treatment, the results provide strong molecular evidence for the participation of both TβRI and Src kinase-containing pathways controlling the transcription of genes involved in eggshell formation in a cooperative and balanced manner.

## Results

### Inhibition of SmTβRI kinase by TRIKI

**Figure 1:**
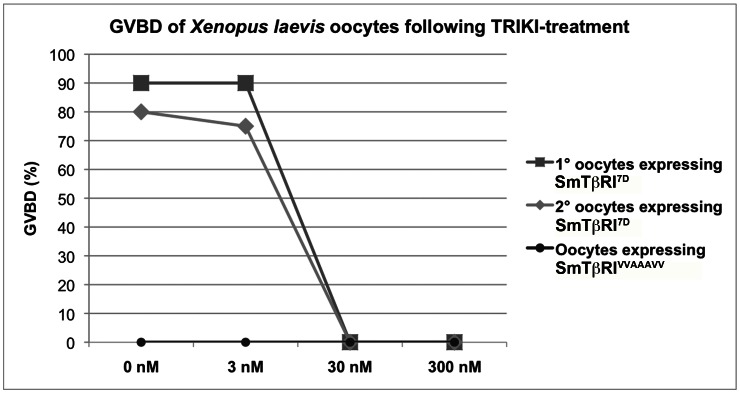
Inhibition of SmTβRI activity by TRIKI. Inhibitory effect of TRIKI on SmTβRI kinase activity was monitored in *Xenopus laevis* oocytes expressing the intracellular domain of SmTβRI by measuring the level of GVBD induced by the kinase in the oocytes. Results of GVBD assays performed in TRIKI-treated oocytes (used concentrations: 0 nM, 3 nM, 30 nM, 300 nM), which expressed either the constitutively active intracellular part of SmTβRI (SmTβRI^7D^; the experiments were performed twice: 1°, dark grey squares; 2°, light grey rhombs), or an inactive kinase variant (SmTβRI^VVAAAVV^, black circles) as control.

The predicted inhibition of SmTβRI by TRIKI (also known as LY-364947) was confirmed by expressing the recombinant intracellular active kinase domain of SmTβRI in *Xenopus laevis* oocytes [30], a suitable system for the expression and detection of kinase activity of schistosome proteins [31]–[34]. In *X. laevis* stage VI oocytes naturally blocked in prophase I of meiosis I, the kinase potential of an exogenous recombinant active kinase triggers resumption of meiosis and thus germinal vesicle breakdown (GVBD), a process easily monitored by the appearance of a characteristic white spot at the animal pole of the oocyte [30]. To functionally analyze the kinase potential of SmTβRI, a constitutively active variant (SmTβRI^7D^) [35] and an inactive one (SmTβRI^VVAAAVV^) were generated by site-directed mutagenesis, and appropriate cRNAs were injected into *Xenopus* oocytes. Results shown in Figure 1 demonstrated that expression of the active SmTβRI^7D^ version induced GVBD in ≥80% of oocytes whereas the inactive one SmTβRI^VVAAAVV^ had no effect on the fate of the oocytes. In the presence of TRIKI, oocytes expressing SmTβRI^7D^ underwent no more GVBD when drug concentrations ≥30 nM were used, confirming the inhibitory effect of TRIKI on SmTβRI kinase. For comparison, previous experiments showed that in this cellular system a complete inhibition of the Src kinase SmTK3 was obtained using 10 nM Herb A [34]. GVBD induced in control oocytes by the natural stimulus progesterone [30] was not affected by TRIKI-treatment (data not shown), demonstrating a specific effect of TRIKI on the SmTβRI kinase in injected oocytes.

### Transcriptome analyses of inhibitor-treated adults by microarrays

Based on previous findings of reduced mitotic activity and egg production following inhibitor treatment of adult female schistosomes *in vitro* [11], [29], we devised an approach to unravel the molecular mechanisms affected by these inhibitors. To this end, large-scale transcriptional analyses were performed using a microarray platform representing nearly the complete *S. mansoni* transcriptome [27], [36], [37]. Our experimental design comprised adult schistosomes that were cultured *in vitro* for 48 h with either TRIKI (300 nM), Herb A (4.5 µM), the combination of both inhibitors (H+T), or with DMSO only as control.

### Identification of genes differentially transcribed following TRIKI-treatment

**Figure 2:**
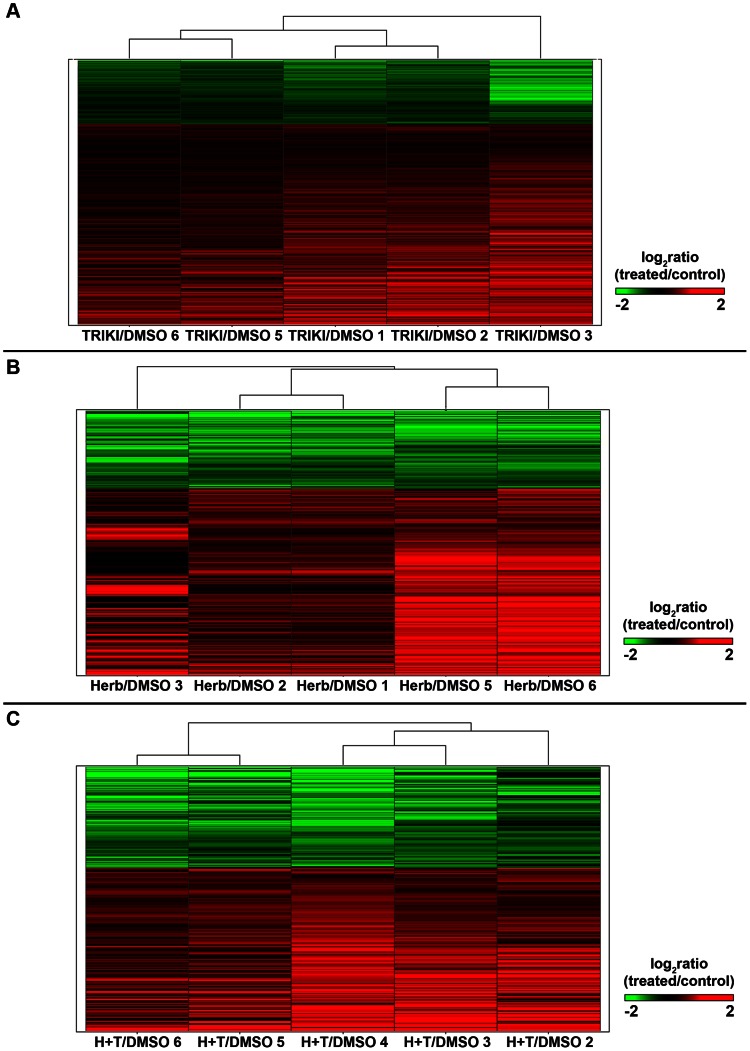
Hierarchical clustering of differentially transcribed genes (q≤0.03) following TRIKI-treatment, Herbimycin A-treatment, and combined inhibitor treatment. Hierarchical clustering of (A) 2330 (TRIKI), (B) 1021 (Herbimycin A), and (C) 411 (combined inhibitors) differentially transcribed genes (q≤0.03) of treated and control female schistosomes. Columns represent three biological replicas as well as two technical replicas (A, TRIKI: columns TRIKI/DMSO 6 and 5, as well as TRIKI/DMSO 1 and 2 represent technical replicas of the first two biological replicas, respectively; TRIKI/DMSO 3 represents the 3^rd^ biological replicate. B, Herbimycin A: columns Herb/DMSO 2 and 1, as well as Herb/DMSO 5 and 6 represent technical replicas of the first two biological replicas, respectively; Herb/DMSO 3 the 3^rd^ biological replicate. C, combined inhibitors: columns H+T/DMSO 6 and 5, as well as H+T/DMSO 4 and 3 represent technical replicas of the first two biological replicas, respectively; H+T/DMSO 2 represents the 3^rd^ biological replicate). Each line shows the calculated log_2_ratio (treated/control) of the transcription of a gene; genes with a repressed transcription were colored in green (A, TRIKI: 565 genes; B, Herbimycin A: 302 genes; C, combined inhibitors: 157 genes) and genes with an enhanced transcription in red (A, TRIKI: 1765 genes; B, Herbimycin A: 719 genes; C, combined inhibitors: 254 genes).

A total number of 8745 genes were detected as expressed in the TRIKI-treatment assays. According to subsequent significance analysis of the microarray data (SAM, q-value≤0.03) 2595 genes were found to be differentially transcribed when compared to the control. Of these, 2330 were protein-coding genes, while 265 had an antisense-orientation relative to the protein-coding gene in the given locus (Supplementary Table S1). A hierarchical clustering of the replicate data was performed (Fig. 2A), showing that the transcription of 1765 protein-coding genes was enhanced (up-regulated, red), and of 565 repressed (down-regulated, green). The opposite tendency was detected for antisense transcripts, where 67 showed enhanced, and 198 repressed transcription. A list with all differentially transcribed genes is available in Supplementary Table S2. Further functional analyses were done only with protein-coding genes.

GO analyses of differentially transcribed genes revealed ontology categories enriched with genes being up- or down-regulated (BH adjusted p-value≤0.05; Supplementary Table S3). The GO category ncRNA metabolic process belonged to the ontology biological process, and contained genes with enhanced transcription. For genes with repressed transcription the ontologies biological process, including the category mRNA metabolic process, and cellular component were detected.

Using IPA, a number of networks enriched with proteins coded by differentially transcribed genes were identified, of which the five most significant are presented (summarised in Supplementary Table S4). The first network included molecules involved in gene expression, protein synthesis, and amino acid metabolism. The second network contained molecules necessary for small molecule biochemistry, lipid metabolism, and amino acid metabolism. Molecules of the third network were associated with cell cycle, cellular function and maintenance, and molecular transport. The fourth network included molecules of nucleic acid metabolism, small molecule biochemistry and DNA replication, recombination and repair. The fifth network consisted of molecules belonging to lipid metabolism, nucleic acid metabolism, and small molecule biochemistry.

### Identification of genes differentially transcribed following Herb A-treatment

A total number of 8016 genes were detected as expressed in the Herb A-treatment assays. SAM identified 1181 genes to be differentially transcribed with a q-value≤0.03. Among these, 1021 represented protein-coding genes and 160 antisense-oriented transcripts (Supplementary Table S5). A hierarchical clustering of the protein-coding genes was performed (Fig. 2B), showing that a majority of 719 genes had an enhanced transcription, whereas 302 exhibited a repressed transcription. The opposite pattern was found for the antisense-oriented genes. Here, 57 genes were enhanced in their transcription and 103 genes repressed (see also Supplementary Table S1). Further functional analyses were done only with probes representing protein-coding genes.

GO analysis resulted in the identification of GO categories (BH adjusted p-value of ≤0.05) significantly enriched only with genes showing enhanced transcription (Supplementary Table S6). The identified categories of the ontology biological process were negative regulation of molecular function, cellular carbohydrate metabolic process, protein folding, and glycoprotein metabolic process. The categories belonging to the ontology molecular function were peptidase regulator activity, enzyme inhibitor activity, and peptidase inhibitor activity. The cellular component ontology included genes belonging to the categories nuclear membrane-endoplasmic reticulum network and endoplasmic reticulum membrane.

Among the five networks detected by IPA as most significantly enriched with proteins coded by genes with altered transcription, network 1 was comprised of molecules related to post-translational modification, protein folding, and molecules known from humans to be involved in cancer (among other signal transduction proteins). The second network included molecules, whose functions are associated in humans with cancer, gastrointestinal disease, and genetic disorder.

For the third network molecules involved in RNA post-transcriptional modification, DNA replication, recombination, and repair and energy production were enriched (Supplementary Figure S1A). The fourth network contained molecules involved in endocrine system development and function, small molecule biochemistry as well as cellular function and maintenance. The fifth network comprised molecules involved in carbohydrate metabolism, cellular function and maintenance, and molecular transport (Supplementary Table S7).

### Identification of genes differentially transcribed following combined Herb A/TRIKI (H+T) treatment

A total number of 11,668 genes were detected as expressed in schistosome parasites in the assays with Herbimycin A and TRIKI. Using SAM statistics, 521 genes were identified as differentially transcribed with a q-value of ≤0.03 (Supplementary Table S8). From these, 411 were protein-coding genes and 110 antisense-oriented transcripts, respectively (Supplementary Table S1). A hierarchical clustering was performed (Fig. 2C), and showed that a higher number of protein-coding genes had an enhanced transcription (254) compared to those with repressed (157) transcription. The same picture was obtained for the antisense messages. Among these, 70 genes showed an enhanced transcription, 40 a repressed transcription upon the combined inhibitor treatment. Further functional analyses were conducted only with protein-coding genes.

By GO analyses a significant enrichment (BH adjusted p-value≤0.05) of genes with an enhanced, as well as repressed transcription was identified for different GO categories (Supplementary Table S9). In summary, enriched genes with enhanced transcription were part of GO categories including multicellular organismal process, localisation of cell, nucleobase, nucleoside, nucleotide and nucleic acid transport, RNA localisation, metabolic process, regulation of cellular metabolic process, regulation of biosynthesis, and cellular nitrogen compound metabolic process (including the corresponding subcategories) for the ontology biological process. The identified categories (and corresponding subcategories) of the ontology molecular function were: hydrolase activity, acting on acid anhydrides, structural molecule activity, binding, and calmodulin binding. Categories comprised of genes showing repressed transcription following the combined inhibitor-treatment included ion binding and peptidase activity, for the ontology molecular function. The ontology biological process included the categories membrane lipid biosynthetic process, protein modification process, and melanin biosynthetic process.

Using IPA, two networks with significantly enriched proteins coded by differentially transcribed genes were identified. The first included molecules associated with post-translational modification, protein folding and cellular comprise. The second network comprised cellular function and maintenance, nucleic acid metabolism, and small molecule biochemistry (Supplementary Table S10).

### Comparative analyses of differentially transcribed genes identified following inhibitor treatments

**Figure 3:**
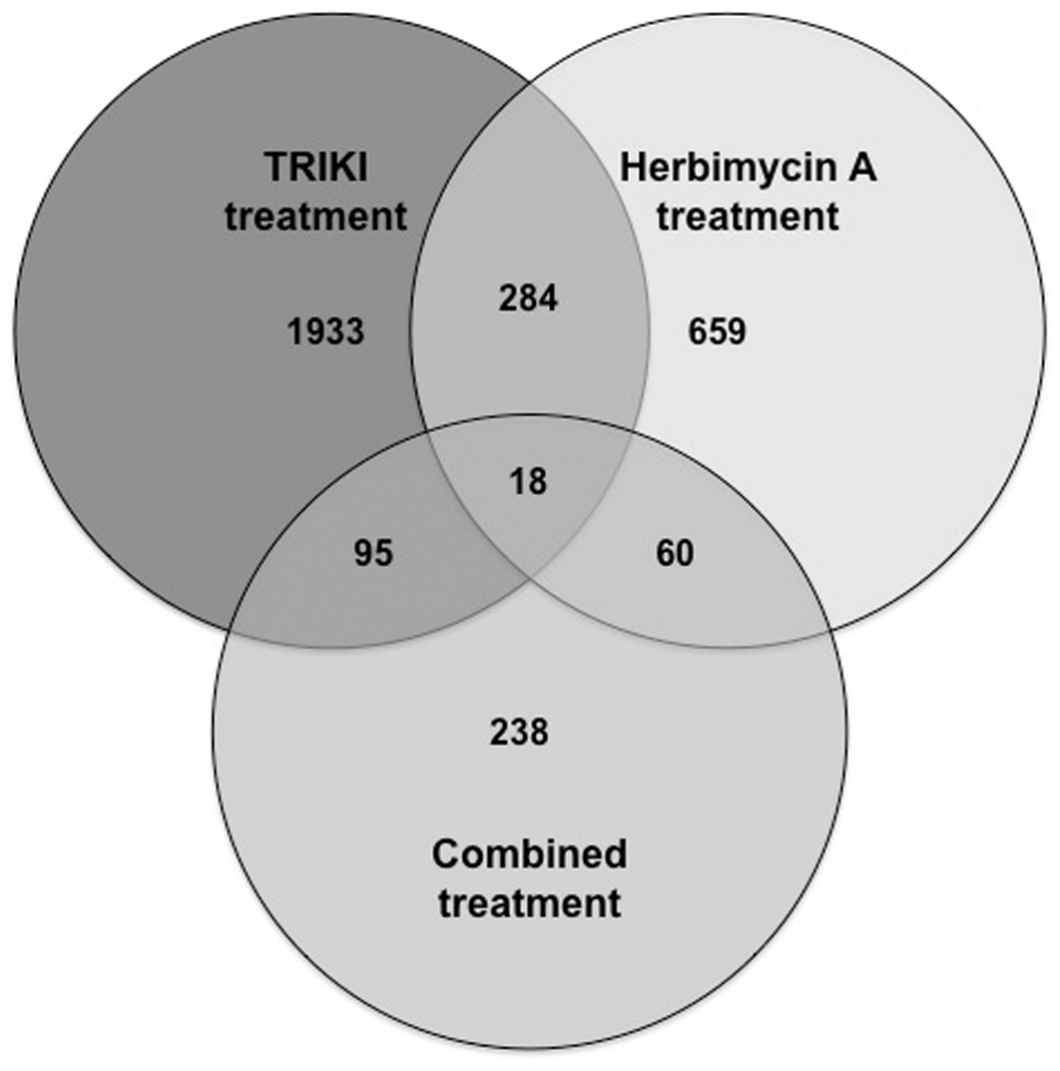
Venn diagram of differentially transcribed genes identified by different inhibitor treatments. Numbers of differentially transcribed protein-coding genes of all three microarray experiments (“TRIKI”, “Herbimycin A”, “Combined treatment”). Each circle represents the microarray result of one of the inhibitor treatments. The intersections show the numbers of corresponding genes differentially transcribed following treatment with different inhibitors.

To get an overview of genes that were influenced by at least one inhibitor treatment, a data comparison was performed analysing the overlapping differentially transcribed protein-coding genes (q≤0.03) and a Venn diagram was created (Fig. 3; Supplementary Table S11).

Transcription of a large number of genes was affected by one treatment only. 1933 genes were exclusively found to be regulated by TRIKI, while the transcription of 659 genes was influenced by Herb A, and the transcription of 238 genes was affected by the combined treatment only. Regulation of 302 genes was affected by TRIKI as well as Herb A, but these genes were not detected as differentially transcribed in the data set from the combined approach. Of these, 87% were transcriptionally regulated in the same direction (213 genes were up- and 50 down-regulated in each of the two treatments), and 13% in inverse directions (18 genes were down-regulated and 21 up-regulated by Herb A treatment, with an opposite pattern in TRIKI treated worms). 113 genes were affected in their transcription by TRIKI or the combined treatment, of which only 32% were equally regulated (34 genes were up- and 2 genes down-regulated in either condition), and 68% of these genes were inversely regulated (13 genes were up- and 64 down-regulated by the combined treatment and showed an opposite change upon treatment with TRIKI alone).

Treatment of worms with Herb A or the combined inhibitors resulted in the differential transcription of 78 genes. In contrast to the proportions of genes whose transcription was influenced by TRIKI-treatment or the combined inhibitors, here 72% were regulated in the same direction (46 genes were up- and 10 genes down-regulated in either condition), and 28% had an inverse pattern (21 genes were up- and 1 gene was down-regulated by the combined treatment and showed an opposite change upon Herb A treatment alone).

The intersection of all three experiments comprised 18 genes, which included e.g. an hsp70-interacting protein, an immunophilin homolog, different hypothetical proteins, and calmodulin-4.

### Validation of selected differentially transcribed genes by quantitative PCR experiments

GO, IPA, and literature-based research were the basis of the selection of differentially transcribed genes, whose inhibitor-influenced transcription was validated by qPCR. Additionally, a manual classification of differentially transcribed genes was used for selecting further candidates.

A huge number of signaling molecules were identified to be affected by at least one inhibitor treatment. Among these were several members of the schistosome TGFβ pathway [12], [21], [38]. Thus two TGFβ superfamily receptors, e.g. SmTβRI (Smp_049760), as well as SmActRIIb (Smp_144390) were transcriptionally enhanced, whereas SmSmad4 (Smp_033950) showed a repressed transcription following TRIKI-treatment. SmActRIIb and SmSmad 4 were transcriptionally regulated in the same direction following Herb A-treatment.

Also genes involved in eggshell formation were found to be influenced by all inhibitor approaches. Selected candidates were the predicted eggshell precursor protein (Smp_000430) and an eggshell protein similar to fs800 (Smp_000270) [39]. Both showed enhanced transcription following TRIKI-treatment, which was unexpected due to the slight negative effect of TRIKI on egg production of female schistosomes shown before [11]. In contrast to this result, the combined treatment led to a repressed transcription for both genes, which was expected according to the strong reduction of the egg production following this dual inhibitor approach [11]. As another eggshell gene Smp48 (Smp_014610) [40] was chosen, whose transcription was repressed following the Herb A-treatment. This inhibitor was shown before to negatively influence egg production of treated couples *in vitro* [11], [29]. Finally, tyrosinase 1 (SmTYR1, Smp_050270) was selected, a gene which was localised in the vitellarium and shown to be responsible for cross-linking processes of eggshell precursor proteins [41]. SmTYR1 was transcriptionally repressed following the combined inhibitory treatment.

As a candidate for surface proteins tetraspanin 18 (Smp_174190) was chosen due to the strongest repressed transcription among the regulated tetraspanins according to TRIKI data analysis. Another member of this protein class was tetraspanin-1 (Smp_011560), which showed the same transcription tendency following Herb A-treatment. Furthermore, the combined inhibitor treatment revealed another tetraspanin 1 homolog (Smp_155310.1) to be transcriptionally repressed. This molecule was also detected within the GO categories membrane and membrane parts. The mentioned tetraspanin homologs have not been characterized in schistosomes yet.

Many heat shock protein genes were identified within the data sets of the individual Herb A- and the combined treatment. Among these was a hsp70 homolog (Smp_106930), which we selected as a representative of transcriptionally enhanced hsps following both inhibitor treatments. Furthermore, IPA of the data sets of both treatments identified hsp70 within network 1, which includes molecules involved in protein folding, in each case. An impact of Herb A on protein folding processes was shown previously to result in the transcriptional activation of hsp70 [42].

Other molecules identified by IPA were the sodium/potassium-pump (Na/K-pump; Smp_015020) and cathepsin S (Smp_139240). As a member of network 3 the Na/K-pump was detected with an enhanced transcription following TRIKI-treatment. Cathepsin S showed a repressed transcription following the combined inhibitor treatment, and it belonged to the network 1. Further candidates were small nuclear ribonucleoprotein (snurp; Smp_069880), which we identified within the GO category “mRNA metabolic process”, and within the IPA network 2 of TRIKI data analysis.

As a representative gene, whose transcription was affected by all three inhibitor treatments, calmodulin-4 (Smp_032990) was selected for validation. It was additionally identified within the category binding of the GO analysis of the combined treatment data set.

**Figure 4:**
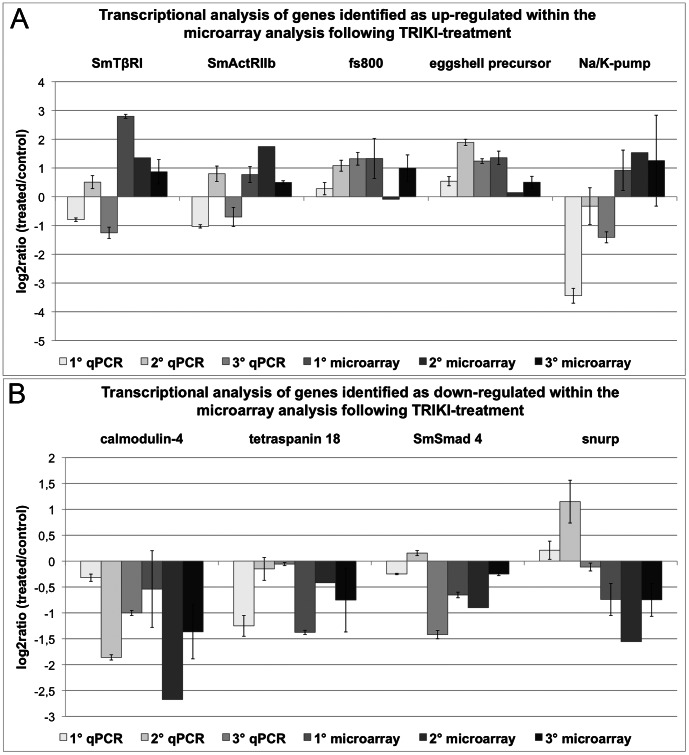
Comparison of qPCR and microarray results following TRIKI-treatment. Transcriptional changes from the TRIKI-treatment of adult schistosome females detected in qPCR and microarray data were calculated as log_2_ratios (treated/control). The investigated genes were SmTβRI, SmActRIIb, a protein similar to fs800, eggshell precursor, and Na/K-pump as representative for enhanced transcription within the microarray data set (A). The selected genes representing the set of genes with repressed transcription within the microarray data set (B) were calmodulin-4, tetraspanin 18, SmSmad 4, and snurp. For each method three biological replicas were used, each with two technical replicas for the microarray analysis (except microarray 2) and three technical replicas for the qPCR. The mean of the technical replicas was calculated and is presented with standard deviations.

For the validation of the microarray data following TRIKI-treatment the genes coding for SmTβRI, SmActRIIb, a protein similar to fs800, a predicted eggshell precursor protein, and Na/K-pump were finally selected as candidates for enhanced transcription (Fig. 4A). Calmodulin-4, tetraspanin 18, SmSmad 4, and a snurp were chosen as representatives of transcriptionally repressed genes (Fig. 4B). In contrast to SmTβRI, SmActRIIb, Na/K-pump, and snurp the results of qPCR and microarray analyses correlated well for all other genes. The different results of the qPCR of both receptors, the Na/K pump, and snurp may be explained by biological variance between worm batches and/or by cross-hybridization. Contradictory results of qPCR and microarray for a small fraction of false-positive genes have already been documented in the literature [43]. Because of the high standard deviations of the microarray log_2_ratio-values, the contradictory results of qPCR and microarray for the Na/K-pump suggested that this gene might belong to the false-positive genes predicted by SAM. Due to the biological variance of the used worm batches, the calculated correlation coefficient according to Spearman was not significant comparing all qPCRs and the corresponding microarray data directly.

**Figure 5:**
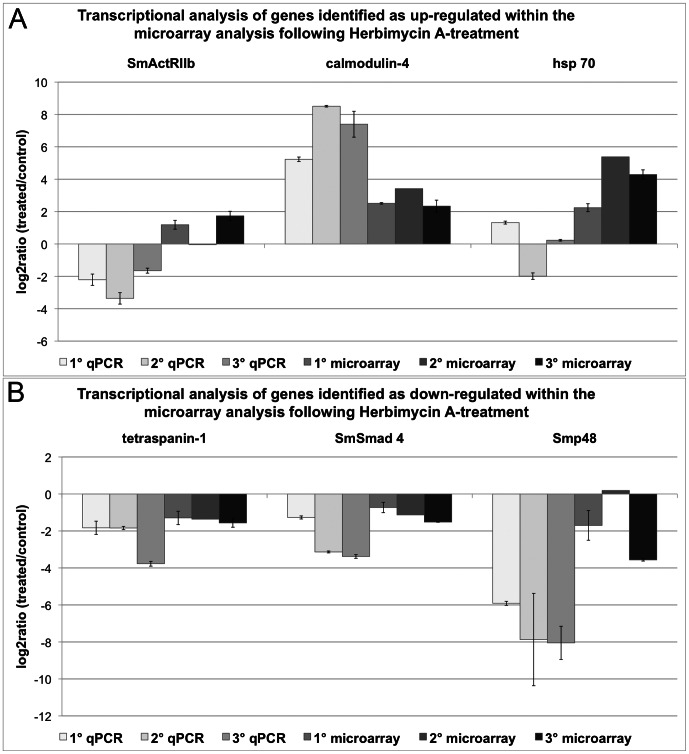
Comparison of qPCR and microarray results following Herbimycin A-treatment. Transcriptional changes from the Herb A-treatment of adult schistosome females detected in qPCR and microarray data were calculated as log_2_ratios (treated/control). According to the microarray analysis SmActRIIb, calmodulin-4, and hsp70 showed enhanced transcription (A), whereas tetraspanin-1, SmSmad 4, and Smp48 were detected as transcriptionally repressed (B). For each method three biological replicas were used, each with two technical replicas for the microarray analysis (except microarray 2) and three technical replicas for the qPCR. The mean of the technical replicas was calculated and is presented with standard deviations.

For the validation of the microarray data following Herb A-treatment the genes coding for SmActRIIb, calmodulin-4 and hsp70 were chosen as representatives for enhanced transcription (Fig. 5A). Tetraspanin-1, SmSmad4 and Smp48 showed a repressed transcription in the microarray data (Fig. 5B). The results of both analyses correlated well for calmodulin-4, hsp70, tetraspanin-1, SmSmad 4 and Smp48. The qPCR result obtained for SmActRIIb was contradictory to that of the microarray, which may have resulted from biological variations of the used worm batches. Nevertheless, the results obtained for these genes with both analyses significant correlated according to Spearman's Correlations Coefficient (r_s_ = 0.886).

**Figure 6:**
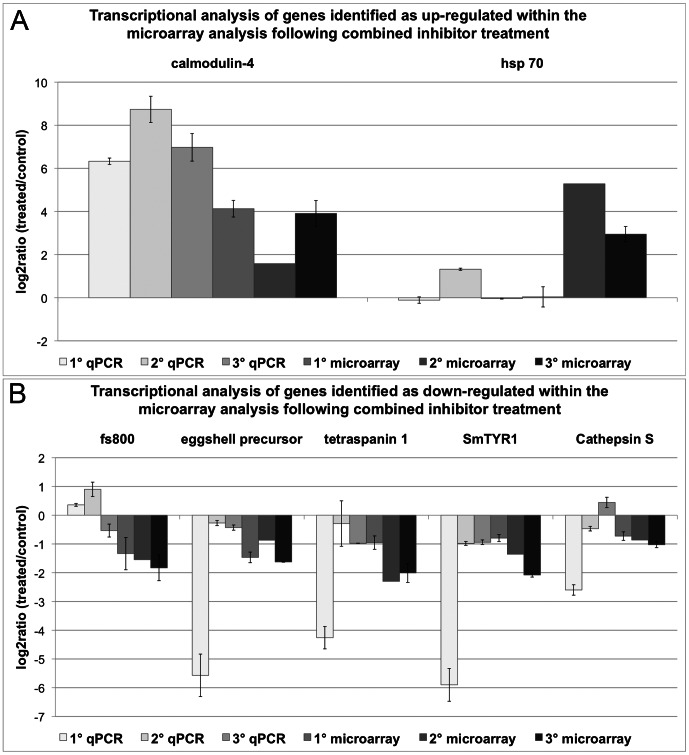
Comparison of qPCR and microarray results following the combined inhibitor treatment. Transcriptional changes from the simultaneous TRIKI- and Herb A-treatment of adult schistosome females detected in qPCR and microarray data were calculated as log_2_ratios (treated/control). The investigated genes with enhanced transcription, as determined by microarray analysis, were calmodulin-4 and hsp70 (A), the genes with repressed transcription comprised a gene similar to fs800, an eggshell precursor protein, tetraspanin 1, SmTYR1, and cathepsin S (B). For each method three biological replicas were used, each with two technical replicas for the microarray analysis (except microarray 2) and three technical replicas for the qPCR. The mean of the technical replicas was calculated and is presented with standard deviations.

The validation of the microarray data of females treated with both inhibitors included the transcriptionally enhanced genes calmodulin-4 and hsp70 (see Fig. 6A). A repressed transcription was detected for the genes coding for a protein similar to fs800, the eggshell precursor protein, tetraspanin 1, SmTYR1 and cathepsin S (Fig. 6B). An increase of transcript levels was confirmed for calmodulin-4 and for hsp70, at least in one qPCR experiment. The identified reduction of transcripts was confirmed for tetraspanin 1, SmTYR1, and cathepsin S. For the gene similar to fs800 an increased transcript level was shown for two biological replicas by qPCR, but for the third qPCR replica and the microarray data a reduction of transcripts was determined. Both results correlated significantly according to Spearman's Correlations Coefficient (r_s_ = 0.7234).

### Transcriptional analyses of selected genes involved in eggshell-formation following inhibitor treatments

During the study we obtained multiple evidence for inhibitor-induced differential transcription of different genes with known function in eggshell formation. In light of this and of previous evidence of a strong negative effect of Herb A as well as a moderate negative effect of TRIKI on egg production [11], functional qPCR experiments were performed focusing on the analysis of a variety of candidates for protein-coding genes involved in this decisive step of the schistosome life cycle. The selected genes were the two well-characterized eggshell precursor protein Smp14 (Smp_131110.x; [44]) and Smp48 (Smp_014610; [40]) as well as a predicted eggshell precursor protein (Smp_000430), which is still uncharacterized. Furthermore, we included the eggshell protein cross-linker SmTYR1 (Smp_050270; [41]) and the eggshell component, which was identified as similar to fs800 (Smp_000270; [39]) into this analysis. Towards this end paired female schistosomes were cultured *in vitro* under the same conditions as before for the microarray approaches to obtain a comparable basis for the transcriptional analyses. For this analysis the microarray data were used independent of their significance values.

**Figure 7:**
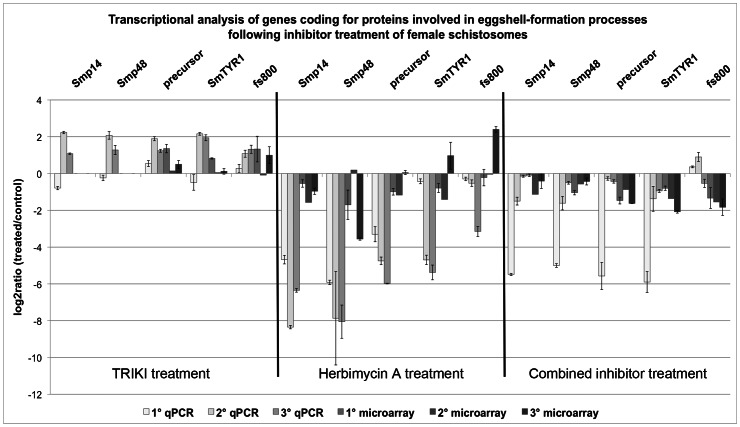
Comparison of qPCR and microarray results of genes involved in eggshell-formation processes. Summary of the log_2_ratio (treated/control) obtained by qPCR and microarray analyses of genes coding for proteins proven or hypothesised to be involved eggshell-formation processes following treatment of paired female schistosomes with either 300 nM TRIKI, 4.5 µM Herb A, or the combination of both inhibitors. The investigated genes were the eggshell precursor proteins Smp14 and Smp48 as well as a predicted eggshell precursor protein (precursor), the tyrosinase 1 (SmTYR1), and a gene similar to fs800. For each method three biological replicas were used, each with two technical replicas for the microarray analysis (except microarray 2) and three technical replicas for the qPCR. The mean of the technical replicas was calculated and is presented with standard deviations.

All three inhibitor treatments influenced the transcription of all genes selected (Fig. 7). In the majority of cases TRIKI-treatment resulted in an increase of transcript levels, which corresponded to the available microarray data. Due to the filtering criteria, the microarray data for Smp14 and Smp48 were not present within the microarray data sets although representative oligonucleotide probes existed. In contrast, SmTYR1 passed filtering during microarray data processing, but the transcriptional changes were not detected to be significant. Significant transcriptional changes were observed by microarrays for the gene similar to fs800 and the eggshell precursor protein following TRIKI-treatment.

For Herb A-treated worms, a reduction of the transcripts of all genes was found by qPCR and this effect was stronger, when compared with the transcriptional changes of the combined inhibitor treatment. Furthermore, the reduced transcription of the qPCR-validated genes in Herb A-treated worms was in accordance with the findings of the microarray analysis. Here a repressed transcription was also determined for Smp14, the eggshell precursor protein, and at least for two biological replicas for SmTYR1 and the protein similar to fs800. The repressed transcription of Smp48 was the only significant transcriptional change within the microarray analysis, although the second replica showed a log_2_ratio of nearly 0.

The combined treatment, finally, led to a repressed transcription of all genes in both analyses, except two biological replicas used for the qPCR analysis for the fs800 gene. Although the tendency of down-regulation of the transcription of the selected genes corresponded well with the results obtained for Herb A-treated females, the effect of the combined treatment was found to be not as strong. Here, the detected decrease of transcripts was significant for the eggshell precursor protein, SmTYR1 and the gene similar to fs800 in the microarray analysis.

In summary, the results confirmed a strong influence of the used inhibitors on transcriptional regulation of chosen genes involved in eggshell-formation processes. Furthermore, the data demonstrated a stronger effect of Herb A compared to TRIKI, which was in accordance with the observation of the physiological effects reported before [11]. Thus the results of our study provide the first molecular evidence that transcriptional regulation of genes involved in eggshell-formation processes is under the control of Src- and TβRI-containing signaling pathways.

### Calcium-associated genes are transcribed in the reproductive system

**Figure 8:**
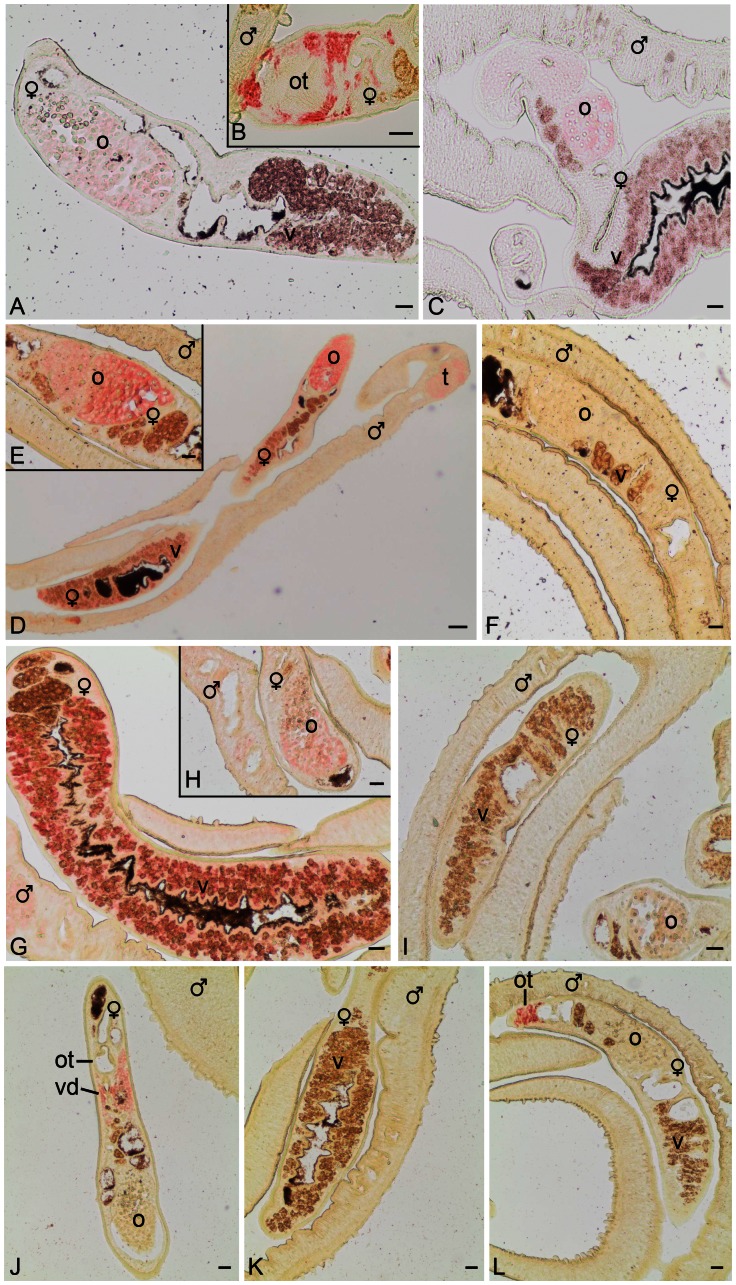
*In situ* hybridization localizing the schistosome homologs of hippocalcin, ORAI-1, a predicted egg-shell precursor protein gene, and calmodulin-4. Sections (5 µm) of adult schistosome couples (males and females are indicated), were hybridized with DIG-labeled antisense-RNA probes of hippocalcin (A, B), ORAI-1 (D, E), eggshell precursor protein gene (G, H), and calmodulin-4 (J, K). For control, DIG-labeled sense-RNAs probes of hippocalcin (C), ORAI-1 (F), eggshell precursor protein gene (I), and calmodulin-4 (L) were used. Signals were observed in the ovary (o), the vitellarium (v), around the ootype (ot) where Mehli's gland is located, and the testes (t). Scale bar: 20 µm.

One additional finding of this study was that a number of genes with predicted calcium-associated functions were among those found to be differentially regulated (see also Supplementary Figure S1B). To provide evidence for their potential contribution to egg production processes we performed localization studies investigating the tissue-specific transcription of candidate genes (Fig. 8). By *in situ* hybridization experiments, transcripts of hippocalcin, a neuronal calcium sensor [45], [46], were detected within the ovary, in the vitellarium and around the ootype (Fig. 8 A, B), where according to classical literature the Mehlis' gland is located [47]–[49]. Transcripts of the potential calcium-influx channel protein ORAI-1 [50] were found to be expressed in ovary and vitellarium of the female and testes of the male (Fig. 8 D, E). Transcripts of the predicted eggshell precursor protein gene were detected within the vitellarium (Fig. 8 G). This was expected with respect to previous findings of the expression of similar genes such as p14 [44], which was used in our study as positive control (data not shown). Additionally, signals of the predicted eggshell precursor protein gene were also observed in the ovary (Fig. 8 H), which was unexpected with regard to p14 that is predominately expressed in the vitellarium. Finally, calmodulin-4 transcripts were observed within the vitelloduct and around the ootype (Fig. 8 J). In this case, the gene prediction indicated a small gene with the consequence that the probe used was relatively short compared to others normally used for this technique. Thus we cannot exclude being close to the detection limit in this case and that calmodulin-4 may be also transcribed in other organs. Furthermore, evidence for the presence of antisense transcripts of hippocalcin (Fig. 8 C) and calmodulin-4 (Fig. 8 L) was found since signals were obtained with sense RNAs in the same tissues. To confirm these transcription patterns, organ-specific RT-PCRs were performed (Supplementary Figure S2) with template RNA of purified testes and ovaries obtained by a novel method for the isolation and enrichment of ovaries and testes [Hahnel et al., submitted]. The results obtained confirmed and complemented the *in situ* findings providing additional evidence for two splice forms of ORAI-1 in testes and ovaries as well as calmodulin-4 transcription in testes.

## Discussion

Inhibitor experiments in previous studies and in this study have indicated that Src kinase- and TβRI-containing pathways influence mitotic activity and egg production in paired schistosome females [11], [29], and that SmTK3 [29], [34] and SmTβRI are targets of Herb A or TRIKI, respectively. Furthermore, a yeast-two-hybrid (Y2H) cDNA-library screening and subsequent qualitative and quantitative analyses identifying and characterizing binding partners acting “downstream” of SmTK3 detected besides others a homolog of the BAF60 subunit of the SWI/SNF complex (SmBAF60) and a diaphanous homolog (SmDia) as the strongest interacting partners [51]. The SWI/SNF complex is involved in chromatin-remodeling activities, DNA-damage responses, transcriptional activation, sliding of nucleosomes, and alteration of histone-DNA contacts [52]. Diaphanous proteins belong to the big group of formin-homology proteins known to play roles in actin-mediated processes controlling cell and tissue architecture, cell-cell interactions, gastrulation, and cytokinesis [53].

To detect genes being controlled by SmTK3- and SmTβRI-containing pathways, presumably playing roles for reproductive processes in adult schistosomes, microarray analyses were performed with RNA of inhibitor-treated paired females as template. A number of genes were detected to be differentially transcribed by individual inhibitors as well as their combination. Among these was a minor amount of antisense RNAs. Besides the possibility that some of these could have protein-coding function, the majority of these RNAs belong to the big group of non-coding RNAs (ncRNAs), and their detection, especially during transcriptome analyzes, has opened a new research field since evidence has accumulated that ncRNAs may have regulatory functions [54]. After first evidence for the occurrence of antisense RNAs in the *S. mansoni* genome was obtained [36], a recently performed detailed analysis estimated that around ≥10% of the transcribed genome may represent non-coding RNAs [37]. According to comparative life-stage analyses, differences in the transcription of schistosome ncRNAs were found indicating their potential roles in diverse biological and physiological processes. Thus it was no surprise to find a fraction of antisense RNAs also in our analysis as being differentially transcribed following treatment with individual or both inhibitors. Among these, some may interfere with regulatory processes. As soon as more knowledge about this class of molecules exists in schistosomes, the findings of our studies may contribute in the future to unravel their function.

The highest number of differentially transcribed genes with protein-coding function was detected for treatment with TRIKI compared to the other treatments. The same tendency was observed when intersections were generated between single treatments and combined treatment, again more genes were found to be differentially transcribed when TRIKI was used. Within the intersection of the individual inhibitor treatments (302 genes; Fig. 3), the majority of differentially transcribed genes were regulated in the same direction, a smaller part in the opposite direction. This indicates that genes within this intersection may be targets of both signaling pathways, which were previously hypothesised to cooperate during cell division and egg production processes [11]. The identity of genes found within this intersection indirectly support this assumption since genes well known for their role in mitosis such as e.g. the cell cycle check point protein rad 17, cyclin 1, or glypican (Supplementary Table S11) were found. Unexpectedly, these genes were all found to be up-regulated by each inhibitor; this was not expected due to the previously observed inhibitory effect of Herb A and TRIKI on mitotic activity [11]. However, other genes contributing to mitosis regulation may have been negatively affected. Indeed, among the genes down-regulated by each inhibitor is a dynactin homolog (Supplementary Table S11). Dynactin is known to direct and coordinate the activities of the dynein motor, which is required for several cellular functions including cell division [55]. Indirect support for these conclusions comes from recent laser-microdissection microscopy (LMM) and oligonucleotide microarray analysis, which detected genes up-regulated two-fold or more in the gastrodermis, the ovary, the vitellarium, and the testes in *S. mansoni* and *S. japonicum* [56], [57]. Among these were rad 17 (ovary, vitellarium), cyclin 1 (ovary), glypican (ovary), and dynactin (ovary, vitellarium) as representatives of genes with elevated transcript levels indicating their functional relevance within the gonads.

Furthermore, an IPA analysis of the combined inhibitor treatment predicted that the c-myc protein might have been activated, since genes theoretically regulated by c-myc were differentially transcribed. Along the same line IPA predicted the protein p53 to be inhibited following Herb A treatment. Since c-myc is able to cause proliferation inhibition (mitoinhibition; [58]) its putative activation may have contributed to the previously observed reduction of mitotic activity following treatment with these inhibitors. Moreover, c-myc expression and p53 inactivation were described as two cell-cycle events regulated by Src during mitosis [59]. Detailed clarifications of these points will be the subject of further studies.

Interestingly, a homolog of Bcl2-associated athanogene (BAG1) was discovered as being up-regulated by each single inhibitor treatment. BAG1 binds to Bcl2, an oncogene inhibiting apoptosis, enhancing its anti-apoptotic effect. In this way BAG1 connects growth factor receptors with anti-apoptotic mechanisms [60]. Indeed, a recent publication provided first evidence that the proliferation of vitelline cells is independent of pairing, but their survival is male-dependent, being pairing-dependently controlled via apoptosis regulation [61], and LMM-microarray analysis showed enhanced Bcl2 transcript-levels in the ovary and the vitellarium [57]. Furthermore, it is noteworthy that a number of genes involved in calcium regulation were differentially regulated such as homologs of hippocalcin [45], [46], [62], [63], different calmodulins [55], or the calcium-influx channel protein ORAI-1 [50]. To provide further evidence for their role in reproduction localization studies were performed demonstrating that the schistosome homologs of hippocalcin, ORAI-1, the predicted eggshell precursor protein gene and calmodulin-4 were transcribed within the reproductive system. Hippocalcin belongs to the calmodulin superfamily and exerts putative sAHP (slow afterhyperpolarization) function in the brain of higher eukaryotes [46]. To our knowledge, there is no information available yet about homologs in invertebrates. Its particular transcription pattern in schistosomes indicates a function of hippocalcin in the ovary, the vitellarium, and within the Mehlis' gland which is known to contribute to egg formation [49]. Since in trematodes Mehlis' glands are connected to the nervous system as shown by the expression of neuropeptides within these glands in *S. mansoni* as well as *Fasciola hepatica* [64], [65], it is tempting to speculate that schistosome hippocalcin may among further functions represent another neuronal player contributing to neurophysiological processes during egg formation. A neurophysiological function was also shown for the calcium channel protein ORAI-1 of *D. melanogaster*, which is required for normal flight and associated patterns of rhythmic firing of the flight motoneurons [66]. In *C. elegans* ORAI-1 knockdown caused complete sterility confirming its essential role in calcium signaling in the gonads [67]. With respect to the localization data obtained in our study, this may apply also to schistosome ORAI-1.

Especially interesting was a calmodulin-4 homolog, which was (i) present in the GO enrichment analysis of Herb A/TRIKI double-treated worms, (ii) a member of the group of 18 genes representing differentially expressed transcripts detected by all three inhibitor approaches, and (iii) seemed to be a target of both pathways being up-regulated following Herb A treatment and down-regulated following TRIKI-treatment as confirmed by our qPCR results (see Figs. 4–6). Links of both pathways to calcium mobilisation and signaling exist. Besides its role as a regulator of the type I TGFβ receptors, the immunophilin FKBP12 regulates the functional state of calcium channel receptors by altering their conformation and coordinating multi-protein complex formation [68]. Among others, Src-kinase signaling can lead to calcium mobilisation contributing to oocyte maturation and fertilisation [69], [70]. A yet uncharacterized immunophilin was also among the 18 differentially transcribed genes identified by all three inhibitor approaches. Although not in each case (ORAI1, no observed up-regulation in the gonads), the above mentioned LMM analysis revealed Ca^2+^-metabolism-associated genes such as hippocalcin (ovary, vitellarium) as transcriptionally enhanced [57]. This and the findings from our study suggest that calcium may also contribute to the physiological processes controlling egg production.

Besides the major intersection of the individual inhibitor treatments, the other intersections between the combined treatment and the individual treatments revealed further groups of genes (113/78; Fig. 3) being representatives of potential targets affected by either TGFβ (113; Fig. 3) or Src pathways (78; Fig. 3). This indicates that one pathway may have a more dominant effect. With few exceptions such as an (yet uncharacterized) eggshell precursor protein within the TRIKI/combined treatment intersection (113; Fig. 3), the majority of both intersections represented hypothetical proteins from *S. mansoni* (Supplementary Table S11), which may be novel, schistosome-specific targets of these pathways. The tendencies of transcriptional regulations between these two groups (113/78) were inversely correlated (group 113: 32%/68% regulated in the same/opposite direction, respectively; group 78: 72%/28% regulated in the same/opposite direction, respectively). We interpret this as another evidence for a stronger response to the inhibition of the Src-kinase containing pathway(s).

A closer look on differentially transcribed genes following TRIKI-treatment revealed a lot of signaling molecules, which included members of the schistosome TGFβ pathway. All type I receptors of TGFβ superfamily were up-regulated by treatment with TRIKI, although only SmTβRI and SmBMPRI were significant for these data, in contrast to the SmActRI. Nevertheless, all three type I receptors showed enhanced transcription, which suggests feedback regulation. This is indirectly supported by the opposite regulation of follistatin, a regulator of TGFβ signaling [25]. It is part of the major overlap of the single inhibitor treatments, being up-regulated by TRIKI and down-regulated by Herb A (302; Fig. 3, Supplementary table S11). Beyond that SmSmad2 and SmSmad4 showed repressed transcription following individual TRIKI or Herb A treatments. The qPCR results indicated some additional influence by biological variability among the worm batches used affecting at least SmTβRI and SmActRI transcription, for which in one of three cases each the same regulatory tendency, as detected in the microarray data, was confirmed. LMM-microarray analysis showed enhanced transcript levels for SmSmad4 (ovary), but not for SmTβRI [56].

A recently published study investigated the transcriptome of adult schistosomes following stimulation with hTGFβ using the same microarray platform [27]. The comparison of the differential transcription of genes upon hTGFβ (∑ 381 genes) or TRIKI-treatment (∑ 1766) revealed 77 genes present in both analyses, of which 58 were regulated in the opposite direction (listed in Supplementary Table S12; Supplementary Figure S3). The higher overall number of differentially transcribed genes of the inhibitor treatment is probably caused by a lower stringency criterion in our study compared to the study of Oliveira et al. [27], in which only genes with a log_2_ratio value ≥|1| were analyzed. Finally, hTGFβ but not BMP7 was shown to be able to bind SmTβRI [26]. Assuming that hTGFβ is not able to activate other type I receptors of the TGFβ superfamily, which is indirectly supported by the identification of the ligands SmBMP [71] and SmInAct [13], the response may be specific leading to a narrow response window. Although previous experiments had shown that TRIKI acts specifically in comparison with others inhibitors (like those of the BMP pathway; [72]), it is nonetheless able to inhibit also TGF-β RII, p38 MAPK, or mixed lineage kinase-7 [73], [74]. Since only homologs of TGFβ RII and p38 MAPK exist in *S. mansoni*, and since inhibiting these would require 10–15 times higher TRIKI concentrations, we expected no alternate target effects. However, we cannot exclude effects on yet unknown targets in schistosomes. Of the 77 genes present in both analyses, 19 genes showed the same regulation pattern. This could be explained by the different sources of RNA used in both studies; whereas in our study RNA of paired females was used, RNA obtained from parasite couples was used in the other study [27]. Since the TGFβ pathway may fulfil different functions in both genders, the regulation of transcription affected by stimulation with hTGFβ can be expected to be different between and within the genders. This could lead to a bias in the transcriptome analyses, which would not be present if both analyses were done with RNA obtained from the same source, e.g. paired females only. Nevertheless, by comparing differentially transcribed genes detected in both treatments, the majority (75%) of genes were found to be affected in the opposite direction, which is in line with the expected outcome of these inversely correlating approaches (correlation of r_s_ = −0,259) (Supplementary Figure S3). The analysis indicated that more genes were transcriptionally repressed than enhanced by hTGFβ, while more genes were enhanced than repressed by TRIKI such as the eggshell protein gene similar to fs800 (Smp_000270; [39]). Herb A-treatment influenced the transcription of different members of the schistosome TGFβ pathway, although not all were significant as defined by our criteria. The homolog of the SmActRIIb belonging to the TGFβ superfamily as well as the SmSmad4 homolog showed the same regulation as observed by TRIKI-treatment (for both homologs a significant influence on the transcription was detected). With respect to the hypothesised pathway cooperation, these findings supportively complement the TRIKI data. Additional support for pathway cooperation was obtained by the Y2H approach identifying SmTK3-interacting molecules, which besides SmBAF60 and SmDia identified a Smad 2/3 homolog as binding partner [51]. SRC/Smad binding during TGFβ-cooperative pathway activities was also found in other cellular systems [75].

**Figure 9:**
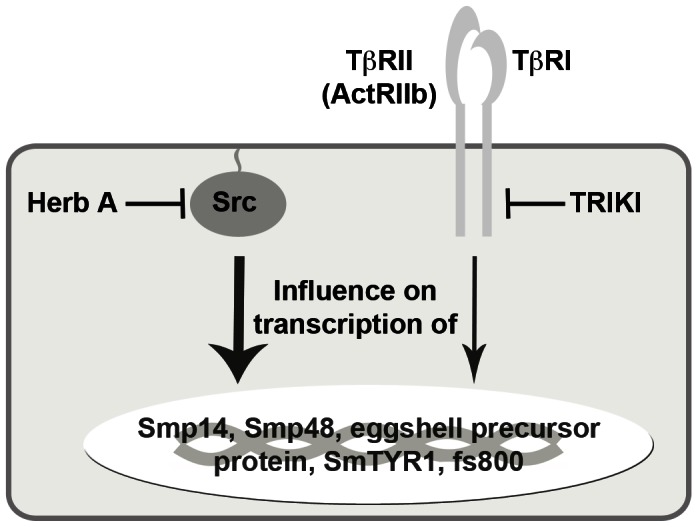
Visual model of the inhibitor effects on transcription of genes involved in eggshell-formation. The inhibition of Src kinases targeted by Herb A and TβRI by TRIKI revealed an influence of both inhibitors on the transcription of genes known to play roles in eggshell formation such as Smp14, Smp48, eggshell precursor protein, SmTYR1 and fs800. Compared to TRIKI treatment, however, a stronger effect on transcription was observed when adult schistosome couples were treated with Herb A. From this we conclude cooperative signal- transduction activities during eggshell-formation with a more dominant role of Src kinases in promoting the expression of involved genes.

The importance of both pathways for egg production in schistosomes was finally confirmed by qPCR experiments focusing on the question whether genes with proven and hypothesised functions for egg formation are influenced in their transcription by these inhibitors. This analysis also included genes from the microarray analysis not high-lighted as significant according to our criteria, such as Smp14. Nonetheless, the qPCR results confirmed enhanced transcription of all genes following TRIKI-treatment, supporting the evidence of a negative influence of the TGFβ pathway on transcription of these genes. This finding was not expected due to the results of a former study, which indicated a slight decrease of egg production upon TRIKI-treatment [11]. However, eggshell formation is a highly complex process and depends on many genes, of which some -yet unknown and/or not represented by this analysis- may be negatively affected by TRIKI-treatment leading to the observed slight decrease in egg numbers. The complexity of egg production-associated processes was also demonstrated by a recent study, in which a direct link between egg production and the mitochondrial oxygen consumption was presented [76]. One of the crucial steps during this process seems to be fatty acid β-oxidation, which is initially catalysed within the mitochondria by acyl CoA dehydrogenase (SmACAD). Within the microarray data obtained for Herb A-treatment, SmACAD transcription was significantly repressed. Since the decreased SmACAD activity was shown to be associated with a decrease of egg production [76], and since SmACAD transcription was negatively influenced by Herb A, we conclude that this gene is also under the control of (a) Src kinase pathway(s). Furthermore, Herb A treatment led to a strong negative effect on the transcription of the genes involved in eggshell formation, which perfectly correlated to the previous finding of a remarkably reduced number of eggs after treatment with this inhibitor. Although the decrease of Smp14 transcription was in contrast to Northern blot data from an elder study [29], which may have been caused by biological (batch) variation, the more sensitive qPCR data obtained here corresponded to the microarray data. Furthermore, all other analyzed genes exhibited the same tendency of transcription regulation. This was also observed following the combined inhibitor treatment. Here, down-regulation of all studied genes was detected, however, it was not as strong as determined for worms treated exclusively with Herb A. From this we conclude that the transcriptional values determined for the combined inhibitor treatment represent an average of the values of both single-inhibitor treatment approaches, which revealed contrary transcription values with a bias towards the Herb A effect. This supports the view of cooperating pathways, but suggests opposing effects of SmTβRI and Src kinase pathways regulating egg production in a balanced way. Concerning the investigated eggshell-forming genes, the influence of both pathways was not equal, since the effect of Herb A dominated that of TRIKI (Fig. 9), which corresponded of the physiological data obtained previously [11]. Although not directly compared in one qPCR experiment, the same tendency was also observed for calmodulin-4 transcription upon treatment with Herb A (strong up-regulation), or TRIKI (weak down-regulation), or both inhibitors (up-regulation). Thus calmodulin-4 may represent another target molecule of the cooperative pathway activity.

The findings presented in this study extend our knowledge on mechanisms controlling reproduction in schistosomes, which is an important, but not yet understood process against the background of understanding basic principles leading to female maturation and egg production in schistosomes and other trematodes. For the first time it is shown that the cooperative actions of TβRI and Src kinase-containing pathways are involved in the control of egg formation, a process not only essential for schistosome life-cycling, but also for the pathological consequences of the disease. In light of the necessity to find alternatives to Praziquantel, the only drug applied worldwide to fight schistosomiasis [77], our findings may also open novel perspectives for alternative concepts to interfere with the transmission of schistosomes by negatively influencing egg production thus interrupting the parasite life cycle. Here kinases have already suggested their potential as targets by *in vitro* experiments [78].

## Materials and Methods

### Parasite stock

A Liberian isolate of *Schistosoma mansoni* was maintained in the intermediate host *Biomphalaria glabrata*, and Syrian hamsters (*Mesocricetus auratus*) as final host [79], [80]. 42 days post infection adult worms were obtained by hepatoportal perfusion.

All animal experiments have been done in accordance with the European Convention for the Protection of Vertebrate Animals used for experimental and other scientific purposes (ETS No 123; revised Appendix A) and have been approved by the Regional Council (Regierungspraesidium) Giessen (V54-19 c 20/15 c GI 18/10).

### *In vitro*-culture and inhibitor treatment of adult schistosomes

After perfusion with M199 medium (Gibco; including glucose, sodium bicarbonate, 4-(2-hydroxyethyl)-1-piperazineethane sulfonic acid), the worms were washed twice with this medium and subsequently cultured in M199 supplemented with FCS (Gibco; 10%), HEPES (Sigma; 1M, 1%), and antibiotic/antimycotic mixture (Sigma; 1%) at 37°C and 5% CO_2_, as described previously [33]. For inhibitor treatments, worm couples were left in culture for 2 days adapting to the *in vitro* environment to restore full egg production capacity [11], [29]. Both Herbimycin A (Herb A, Enzo Life Science; CAS: 70563-58-5; 1 mg/ml) and the TβRI kinase inhibitor (TRIKI, or LY-364947; purchased from Calbiochem; CAS: 396129-53-6; 5 mg/ml) were dissolved in dimethyl sulfoxide (DMSO). TRIKI (C_17_H_12_N_4_) is a pyrazole-based inhibitor (3-(pyridin-2-yl)-4-(4-quinonyl)-1H-pyrazole) with an IC_50_ value of 51 nM for human TβRI (Calbiochem; [73], [74]). It binds to the active site of the TβRI kinase domain and is less effective inhibiting TGF-β RII (IC_50_ = 400 nM), p38 MAPK (IC_50_ = 740 nM), or mixed lineage kinase-7 (MLK-7; IC_50_ = 1,400 nM).

For transcriptome studies the cultivation of adult schistosomes was done for 2 days with either 4.5 µM Herb A, 300 nM TRIKI, or the combination of both inhibitors (H+T) with the same concentrations. As control, worms were cultivated in medium containing DMSO. Medium and additives were refreshed daily. Pairing stability and vitality were checked each day. As vital and useful for further experiments we considered couples, whose males sucked with their ventral suckers to the Petri dish, while keeping the female within the gynaecophoric canal. Vital worms performed uniform, wave-like movements, showed regular gut peristaltic, and produced eggs. Separation of couples and/or failing of males to suck to the Petri dish, resting on the side and showing reduced wave-like movements were considered as signs of decreasing vitality. Such worms were removed from the dishes and not considered for further analyses.

Worms used for the experiments (treated and control samples likewise) were carefully separated by pipetting or using featherweight tweezers, immediately shock-frozen in liquid nitrogen, and stored at −80°C for further use.

### GVBD assays in *Xenopus* oocytes

To test whether TRIKI was able to inhibit SmTβRI (Smp_049760), its intracellular part was cloned into the expression vector pcDNA 3.1/V5-His B. To this end this region was amplified by PCR with cDNA as template and the primers TGFβRI_intra_*Bam*HI-5′ (5′-GGATCCTACTTCCTCTGGAGAAGGAAATC-3′) and TGFβRI_intra_*Eco*RV-3′ (5′-GATATCTAAATGCTTTGAATTACTATTGTTATTGG-3′). Both primers contained specific restriction sites (5′ primer *Bam*HI; 3′ primer *Eco*RV), which were used for directed cloning of the amplification product into the vector. The obtained wild type (wt) SmTβRI-pcDNA 3.1/V5-His construct was commercially sequenced (LGC Genomics, Berlin) to confirm the correct open reading frame (ORF). To convert wt SmTβRI into a constitutively active variant, the Ser and Thr residues of the GS motif (position 284–290) as well as the Thr (position 299) and Gln (position 303) were mutated into 7 Asp (SmTβRI^7D^) as described earlier [35]. For negative control, an inactive kinase variant was generated by exchanging Thr residues to Val, as well as by exchanging Ser and Gln residues to Ala (SmTβRI^VVAAAVV^). These mutations were done successively by site-directed mutagenesis, using the SmTβRI-pcDNA 3.1/V5-His wt construct as template (25 ng) and the following primers: TGFβRI_Mut-1(5D)-5′ (5′-GATGGACCACGATGACGATGGGGACGGTGACGGAAAACCTTTACT-3′) + TGFβRI_Mut-1(5D)-3′ (5′-AGTAAAGGTTTTCCGTCACCGTCCCCATCGTCATCGTGGTCCATC-3′); TGFβRI_Mut-2(7D)-5′ (5′-CCTTTACTAGTTCAGCGAGATGTCGCTAGGGACGTTCAGTTGG-3′) + TGFβRI_Mut-2(7D)-3′ (5′-CCAACTGAACGTCCCTAGCGACATCTCGCTGAACTAGTAAAGG-3′); TGFbRI_Mut-1(VVAAA)-5′ (5′-GATGGACCACGTTGTCGCTGGGGCAGGTGCCGGAAAACCTT-3′) + TGFβRI_Mut-1(VVAAA)-3′ (5′-AAGGTTTTCCGGCACCTGCCCCAGCGACAACGTGGTCCATC-3′); TGFβRI_Mut-2(VV)-5′ (5′-CCTTTACTAGTTCAGCGAGTGGTCGCTAGGGTAGTTCAGTTGG-3′) + TGFβRI_Mut-2(VV)-3′ (5′-CCAACTGAACTACCCTAGCGACCACTCGCTGAACTAGTAAAGG-3′). For PCR the proofreading *Pfu* DNA-Polymerase (Promega; 3 U/µl) was used followed by *Dpn*I digestion for 1 h at 37°C to digest remaining wt vector DNA [81]. Following bacteria transformation and plasmid-DNA isolation, sequencing confirmed the correct ORFs of the constructs. Subsequently, cRNA synthesis was done as previously described [32], and 60 ng each used for microinjection into *Xenopus laevis* stage VI oocytes [31], [32]. The oocytes were cultured in ND96 medium at 19°C for 18 h. Germinal vesicle break down (GBVD), a witness of meiosis progression dependent on kinase activity [30], is characterized by the development of a white spot at the animal pole of the oocyte. As a positive control for GVBD, oocytes were stimulated with progesterone. As negative controls, non-injected oocytes as well as oocytes transfected with the inactive kinase variant SmTβRI^VVAAAVV^ were used. For inhibitor studies, oocytes were cultured in ND96 medium supplemented with different concentrations of TRIKI (3 nM, 30 nM, and 300 nM) for 18 h.

### RNA extraction and microarray experiments

RNA from inhibitor-treated or from control females was isolated using Trizol reagent (Invitrogen). Subsequently, a DNAse digestion was done with the RNAeasy kit (Qiagen) according to the manufacturer's manual. The quality of the isolated RNA was determined using Bioanalyzer microfluidic electrophoresis (Agilent Technologies).

For microarray experiments a *S. mansoni* custom-designed oligonucleotide platform (60-mers) was used, with approximately 44,000 probes representing nearly the complete *S. mansoni* transcriptome based on available cDNA sequence data from *S. mansoni* and *S. japonicum*. This platform was produced by Agilent Technologies (described in [36], and all associated information (probes, annotation) is available at Gene Expression Omnibus (GEO) under the accession number GPL8606.

From each sample of the three inhibitor treatments, 300 ng RNA were used for cDNA amplification followed by Cy3 and Cy5 labelling during *in vitro* transcription using the Quick Amp Labelling Kit, two colors (Agilent Technologies). Labelling included a dye-swap approach as an internal technical replica for each sample. Thus, six microarray hybridizations were performed per inhibitor treatment and corresponding controls, including two technical replicas for each of the three biological replicas. For hybridization, 825 ng cRNA of each labelled inhibitor sample were used and combined with a control sample labelled with the opposite dye.

Hybridization was done at 65°C for 17 h with rotation. The slides were washed and scanned with the Gene Pix 4000B Scanner (Molecular Devices) according to the Agilent manual. The obtained raw data were extracted using Feature Extraction software (Agilent Technologies) and are available under GEO study number GSE39732. For subsequent analyses, a gene was considered as expressed only if its corresponding probe exhibited signals that were significantly higher than background (employing default parameters from the Feature Extraction software and recovering the column “IsPosAndSig” from the output). A probe had to fulfil the criterion to have detectable expression in at least 75% of all replicas in at least one of the two conditions (inhibitor-treated or control). LOWESS algorithm was used for normalisation of the intensities [82], and the log_2_ratios between inhibitor-treated and control groups were calculated. Subsequently, an adjustment of these filtered data was done using an updated genome annotation to eliminate redundancy of the probes per gene [37]. A low overall correlation was observed for the technical replicates Herb/DMSO 4 and H+T/DMSO 1, leading to the exclusion of these experiments from further analyses. As a consequence, the technical replicate TRIKI/DMSO 4 treatment was removed additionally to achieve a comparable basis for the analysis of all three microarray approaches; the latter technical replicate was selected for exclusion based on its lowest total intensities of the fluorescence signals.

To identify genes with a significant change in the levels of transcribed message, SAM (Significance Analysis of Microarrays) was used [83]; genes with a q-value≤0.03 were considered to have a significantly differential level of transcribed message between inhibitor-treated and control samples. The subsequent functional analyses focused on probes representing protein-coding genes (labelled as “to be used in GO = YES” in the updated annotation of the array [37]), although antisense-oriented oligonucleotide probes were present on the microarray platform as well. Hierarchical clustering was done using Spotfire [84]. Functional analyses were performed for differentially transcribed genes; Gene Ontology (GO) analyses [85] of all three inhibitor data sets were done with the software tool Ontologizer [86]; parent child union [87] was selected to identify categories containing enriched genes, and the p-value was adjusted according to Benjamini-Hochberg (BH) correction [88]. Further functional analyses were performed by Ingenuity Pathway Analysis (IPA; http://www.ingenuity.com; [89]), which represents a tool providing curated information from the literature for human, mouse and rat models about canonical pathways, regulated transcription factors and their targets, and possibly regulated molecular networks, including signal transduction cascades (of which some contribute to human cancer and other diseases). To this end, all *S. mansoni* genes were annotated with the corresponding human homolog (determined according to a search with the following blast parameters: e-value<10^−10^ and at least 60% coverage); genes that fulfilled these criteria were annotated with the label “to be used in IPA = YES” in the updated annotation [37], and they were uploaded to IPA along with their corresponding microarray transcription measurements. These putative homologs represented the basis for the identification of pathways and networks enriched with proteins encoded by significantly differentially transcribed genes; default settings were used, except for presentation of networks (140 molecules per network, number of networks: 10) and for “user dataset” as reference set.

### Quantitative PCR experiments

To validate the transcriptional changes caused by inhibitor treatment of selected genes quantitative PCRs (qPCRs) were performed on a Rotor Gene Q (Qiagen). RNAs from inhibitor-treated or control females were isolated using TriFast reagent (PeqLab), and 1 µg each was reverse transcribed using QuantiTect Rev. Transcription kit (Qiagen) according to the manufacturer's instruction. The amplified cDNA was diluted 1∶20 and used for subsequent qPCR analyses. The detection of synthesised DNA double strands was based on the incorporation of SYBRGreen using PerfeCTa SYBR Green Super Mix (Quanta). To distinguish between the specific amplification product and unspecific primer dimers following each qPCR analysis a melting point analysis was done. Primer 3 Plus software was used for primer design (http://www.bioinformatics.nl/cgi-bin/primer3plus/primer3plus.cgi). The amplification products had a size between 140 and 160 bases. Primers were designed to have melting points at 60°C. Furthermore, differentiation of amplification products of cDNA and genomic DNA was obtained by designing primers that flanked predicted introns of the appropriate genes. A list of all used primers is available in Supplementary Table S13. All primers were commercially synthesised by Biolegio (Netherlands).

Since standard reference genes normally used for relative quantification analyses such as α-tubulin, Cu/Zn SOD (superoxide dismutase), or histone showed regulation following inhibitor treatment, we decided to perform absolute quantification on the basis of standard curves generated by purified PCR products (used in dilution series) [90]. These quantifications were done for both inhibitor- and DMSO-treated samples. Fold changes are available in Supplementary Tables S2, S5, and S8. Subsequently, log_2_ratios (treated/control) were calculated according to a previous study providing a solid basis for comparison of microarray and qPCR data [91]. The efficiency of each qPCR was determined to be between 90–100%. Finally, the validity of the obtained ratios of qPCRs and microarrays was determined by Spearman's rank correlation coefficient (r_s_) as well as the correlation of the intersection of the microarray data following hTGFβ and TRIKI-treatment [92], [93].

### *In situ* hybridization experiments

To detect the occurrence of transcripts of ORAI-1, hippocalcin, the predicted eggshell precursor protein gene, and calmodulin-4 of *S. mansoni*, *in situ* hybridizations were performed as described earlier [33], [51]. To this end, adult worm pairs were fixed in Bouin's solution (picric acid/acetic acid/formaldehyde; 15/1/5) and embedded in paraplast (Histowax, Reichert-Jung) before sections of 5 µm were generated and incubated in xylol. Following re-hydration, proteins were removed by proteinase K treatment (freshly prepared, final concentration 1 µg/ml), and the sections were dehydrated. For hybridization, transcripts were generated *in vitro* by RT-PCR, checked for their identity by sequencing, and labeled with digoxigenin following the manufacturers' instructions (Roche). The following primer combinations were used for amplification (hippocalcin, Smp_085650: forward 5′-GCTATTTATGCGATGGTTGGC-3′, reverse 5′-GACTCTGAGGTATCAGGAATGAC-3′; ORAI-1, Smp_076650.1: forward 5′-GTTGTCGTGCATATAATGGCT-3′, reverse 5′-CTGGACTCCACTTCTAAGAAAGG-3′; eggshell precursor protein gene, Smp_000430: forward 5′-GTTCCAATTACCAACCAACGTC-3′, reverse 5′-GTTTCCGTTACCACCATAATTACC-3′; calmodulin-4, Smp_032990: forward 5′-ATGAATGTTCCAATAACTCGTGAAG-3′, reverse 5′-AAGTGCTCTTGTTAATTCTGGTAAAC-3′). Primers were 5′-tagged by the addition of the T7-sequence (5′-TAATACGACTCACTATAGGGAGA-3′) to allow RT-PCR-based product synthesis of antisense or sense probes using T7 polymerase (Roche). PCR conditions to amplify calmodulin-4 were: denaturation 95°C 45 s, annealing 56°C 45 s, elongation 72°C 45 s; 30 cycles; for all other transcripts: denaturation 95°C 45 s, annealing 60°C 45 s, elongation 72°C 30 s; 30 cycles. Labeled transcripts of hippocalcin (359 bp), ORAI-1 (428 bp), the hypothesized eggshell precursor protein gene (533 bp), and calmodulin (210 bp) were size-controlled by gel electrophoresis. To prove their quality, transcript blots were made confirming digoxigenin-incorporation by alkaline phosphatase-conjugated anti-digoxigenin antibodies (Roche) with naphthol-AS-phosphate and Fast Red TR (Sigma). All *in situ* hybridizations were performed for 16 h at 42°C. Sections were washed up to 1× SSC, and detection was achieved as described for transcript blots.

### RT-PCR with organ-specific RNA

Testes and ovaries were isolated by a recently established organ-isolation method [Hahnel et al., submitted]. In short, adult males or females (50–60 each) were treated with 500 µl of tegument solubilisation (TS)-buffer (0.5 g Brij35, 0.5 g Nonidet P40, 0.5 g Tween80, and 0.5 g TritonX-405 per 100 ml PBS (137 mM NaCl, 2.6 mM KCl, 10 mM Na_2_HPO_4_, 1.5 mM KH_2_PO_4_ in DEPC-H_2_O, pH 7.2–7.4)) at 37°C and 1,200 rpm in a shaker for 5 min to solubilise the tegument. This step was repeated for females (1×) and males (2×) followed by washing steps (3×) with M199 medium (2 ml). To remove the muscles, elastase IV from pancreas (Sigma, #E0258) was used (5 units/ml, in M199 medium), and 500 µl added to each sample followed by slight agitation (600 rpm) in a shaker at 37°C for about 30 min. During incubation, the worms were swirled up manually every 5 min. This reaction was stopped when the medium turned opaque and the worms were fragmented but not completely digested. At this point liberated intact organs were observed. Testes and ovaries were identified by their characteristic morphology and carefully transferred by pipetting to fresh M199 medium. For quality inspection bright field microscopy was performed and if necessary, the organs were separated from remaining tissue rests by additional washes and transfers. Finally, the organs were collected by a pipette, transferred to 1.5 ml tubes, and concentrated by centrifugation for 5 min at 1,000 g (testes) or 1 min at 8,000 g (ovaries). After removal of the supernatant the organs were frozen in liquid nitrogen and stored at −80°C for further use.

For cDNA synthesis the QuantiTect Reverse Transcription Kit (Qiagen) was used with 500 ng of total RNA as template following the instruction of the manufacturer. PCR was done with 2 µl of a 1∶40-dilution (testis cDNA) or 1∶80-dilution (ovary cDNA) of each cDNA-sample in a total volume of 50 µl containing 1× reaction buffer (80 mM Tris-HCl, 20 mM (NH_4_)_2_SO_4_, 0.02% w/v Tween20, 2.5 mM MgCl_2_), 200 µM dNTPs, 400 nM of each primer and 2.5 units Fire-Pol *taq* polymerase (Solis BioDyne). The reactions were performed in a MasterCycler (Eppendorf) programmed as follows: 1 cycle, 95°C, 2 min; 35 cycles, 95°C, 45 sec; 60°C, 45 sec; 72°C, 45 sec. The primers used to amplify hippocalcin, eggshell precursor protein gene, and calmodulin-4 were mentioned above, primers used for ORAI-1 amplification were newly designed to be able to detect both splice variants of this gene (Smp_076650.1, Smp_076650.2; forward 5′- ACGTTGTTACTTCTTCAGTACTCC-3′; reverse 5′-ACTTTGTAGGTAGTAAGCGCAC-3′). As positive control for similar amounts of cDNA of each organ-sample the *S. mansoni* heat shock protein 70 gene (SmHSP70 accession number L02415; forward 5′-TGGTACTCCTCAGATTGAGGT-3′; reverse 5′-ACCTTCTCCAACTCCTCCC-3′) was used since as it was described to be expressed throughout diverse life stages and tissues, and it turned out to be a suitable control [94; Hahnel et al., submitted].

### In silico analysis

The following public domain tools were used: SchistoDB (http://schistodb.net/schisto/), Gene DB (http://www.genedb.org/Homepage), Welcome Trust Sanger Institute *S. mansoni* OmniBlast (http://www.sanger.ac.uk/cgi-bin/blast/submitblast/s_mansoni/omni), BLAST (http://blast.ncbi.nlm.nih.gov/), Clustal W2 (http://www.ebi.ac.uk/Tools/msa/clustalw2/), InterPro (http://www.ebi.ac.uk), SMART (http://smart.embl-heidelberg.de/).

### Abbreviations

TRIKI, TβRI kinase inhibitor; Herb A/Herb, Herbimycin A; H+T, Herbimycin A combined with TRIKI, TGFβ, transforming growth factor beta; hTGFβ, human TGFβ; SmTβRI, *S. mansoni* type I TGFβ receptor; SmActRIIb, *S. mansoni* type IIb Activin receptor; SmSmad, *S. mansoni* Smad; SmTYR1, *S. mansoni* tyrosinase 1; SmTK3, *S. mansoni* tyrosine kinase 3; SmFKBP12, *S. mansoni* FK506-binding protein 12; fs800, female specific protein 800; ORAI-1, calcium release-activated calcium channel protein 1; qPCR, quantitative PCR; CLSM, confocal laser scanning microscopy; GVBD, germinal vesicle break down; SAM, Significance Analysis of Microarrays; GO, Gene Ontology; IPA, Ingenuity Pathway Analysis; BH, Benjamini-Hochberg correction; snurp, small nuclear ribonucleoprotein; hsp, heat shock protein

## Supporting Information

Figure S1 **Network 3 of the IPA analysis following Herbimycin A-treatment (q≤0.03).** Example of an IPA-based network (no. 3) presenting proteins coded by differentially transcribed genes following Herb A-treatment. These molecules are involved in RNA post-transcriptional modification, DNA replication, recombination as well as repair and energy production. The shapes of the genes correlate with the functional classification symbolised in the legend. A = entire network. B = close up of the framed area in A (square). Arrows represent the relationship between molecules: dashed lines = indirect interaction, continuous lines = direct interaction; color intensity correlates to transcription value, calculated as log_2_ratio (treated/control) in Herb A-treated paired females; green represents molecules with repressed transcription (negative log_2_ratio); red represents molecules with enhanced transcription (positive log_2_ratio); grey represents molecules present in the dataset, but did not meet the defined cut-off for differential transcription; white represent molecules, which were included in the network because of their known relationships with other detected molecules, but they were not present within the data set.
(TIF)

Figure S2 **RT-PCR with organ-specific RNA confirmed and complemented** ***in situ*** **-hybridization data.** Shown are RT-PCR results with organ-specific RNA of purified testes (lanes 1, 3, 5, 7, 9) and ovaries (lanes 2, 4, 6, 8, 10) indicating the presence of transcripts of hippocalcin (lanes 1, 2), ORAI-1 (lanes 3, 4), the egg-shell precursor gene (lanes 5, 6), and calmodulin-4 (lanes 7, 8). As positive control hsp70 was used (lanes 9, 10), which was shown to be widely expressed [94] serving as a suitable control also for gonad tissue [Hahnel et al., submitted]. The two bands in lanes 3 and 4 resulted from two different splice forms of ORAI-1 (Smp_076650.1, Smp_076650.2; expected products 491 bp and 532 bp), which were detected both by the used primers. M: HyperLadder (Bioline).
(TIF)

Figure S3 **Comparison of differentially transcribed genes following hTGFβ- or TRIKI-treatment.** Hierarchical clustering of genes differentially transcribed after either hTGFβ stimulation [27] or TRIKI-induced inhibition of female schistosomes. Each line represents one of 77 genes that were identified to be differentially transcribed in both microarray analyses. The comparison was done using the mean log_2_ratio (treated/control) of transcription of these genes; genes with an enhanced transcription in treated compared to control are shown in red, and with a repressed transcription in green.
(TIF)

Table S1 **List of the numbers of significantly differentially transcribed genes following inhibitor treatments (q≤0.03).** This table contains a list of the numbers of significantly differentially transcribed genes following inhibitor treatments (q≤0.03).
(DOCX)

Table S2 **List of genes found to be significantly differentially transcribed following TRIKI-treatment.** This list contains all information about genes found to be significantly differentially transcribed following TRIKI-treatment (including subdivisions of up- and down-regulated sense and antisense transcripts).
(XLSX)

Table S3 **Gene Ontology categories enriched with differentially transcribed genes following TRIKI-treatment.** Gene Ontology categories are listed enriched with differentially transcribed genes following TRIKI-treatment (including up- and down-regulated categories).
(XLSX)

Table S4 **Networks of enriched molecules identified by IPA using the data set of differentially transcribed genes following TRIKI-treatment.** This is a list of networks of enriched molecules identified by Ingenuity Pathway Analysis (IPA) of genes differentially transcribed following TRIKI-treatment.
(XLSX)

Table S5 **List of genes found to be significantly differentially transcribed following Herbimycin A-treatment.** This list contains all information on genes found to be significantly differentially transcribed following Herbimycin A-treatment (including subdivisions of up- and down-regulated sense and antisense transcripts).
(XLSX)

Table S6 **Gene Ontology categories enriched with significantly differentially transcribed genes following Herbimycin A-treatment.** Gene Ontology categories are listed enriched with differentially transcribed genes following Herbimycin A-treatment (including up-regulated categories).
(XLSX)

Table S7 **Networks of enriched molecules identified by IPA using the data set of differentially transcribed genes following Herbimycin A-treatment.** This is a list of networks of enriched molecules identified by Ingenuity Pathway Analysis (IPA) of genes differentially transcribed following Herbimycin A-treatment.
(XLSX)

Table S8 **List of genes found to be significantly differentially transcribed following combined treatment with Herbimycin A and TRIKI.** This list contains genes found to be significantly differentially transcribed following combined treatment with Herbimycin A and TRIKI (including subdivisions of up- and down-regulated sense and antisense transcripts).
(XLSX)

Table S9 **Gene Ontology categories enriched with significantly differentially transcribed genes following combined treatment with TRIKI and Herbimycin A.** Gene Ontology categories are listed enriched with differentially transcribed genes following combined TRIKI and Herbimycin A-treatment (including up-regulated categories).
(XLSX)

Table S10 **Networks of enriched molecules identified by IPA using the data set of differentially transcribed genes following combined treatment with TRIKI and Herbimycin A.** This is a list of networks of enriched molecules identified by Ingenuity Pathway Analysis (IPA) of genes differentially transcribed following combined TRIKI and Herbimycin A-treatment.
(XLSX)

Table S11 **Intersections of genes differentially transcribed following treatment with TRIKI and Herbimycin A.** This list contains all intersections from the single as well as the combined treatments with TRIKI and/or Herbimycin A (4 subdivisions).
(XLSX)

Table S12 **Intersection of significantly differentially transcribed genes following hTGFβ stimulation and TRIKI-treatment.** This list contains the intersection of significantly differentially transcribed genes following hTGFβ stimulation and TRIKI-treatment subdivided in “overlap”, “different regulation”, and “same regulation” categories.
(XLSX)

Table S13 **List of primers used for qPCR.**
(DOCX)
