# Supplementary figures and images for: Transcriptome Analyses of Inhibitor-treated Schistosome Females Provide Evidence for Cooperating Src-kinase and TGFβ Receptor Pathways Controlling Mitosis and Eggshell Formation
Source: PLoS Pathog. 2013 Jun 13;9(6):e1003448. doi: 10.1371/journal.ppat.1003448 (PMC3681755; doi:10.1371/journal.ppat.1003448)

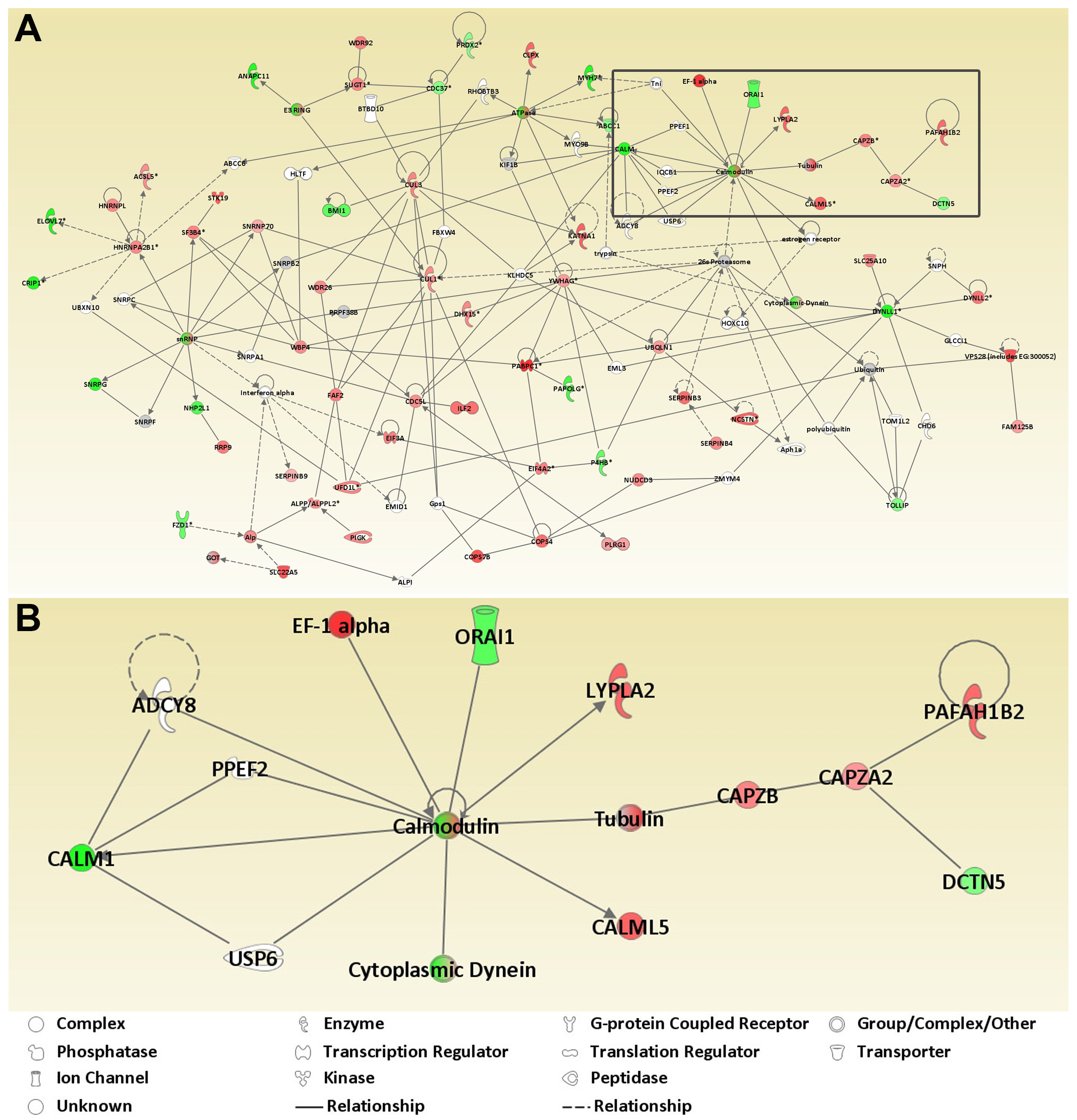

Supplement: Figure S1 — Network 3 of the IPA analysis following Herbimycin A-treatment (q≤0.03). Example of an IPA-based network (no. 3) presenting proteins coded by differentially transcribed genes following Herb A-treatment. These molecules are involved in RNA post-transcriptional modification, DNA replication, recombination as well as repair and energy production. The shapes of the genes correlate with the functional classification symbolised in the legend. A = entire network. B = close up of the framed area in A (square). Arrows represent the relationship between molecules: dashed lines = indirect interaction, continuous lines = direct interaction; color intensity correlates to transcription value, calculated as log2ratio (treated/control) in Herb A-treated paired females; green represents molecules with repressed transcription (negative log2ratio); red represents molecules with enhanced transcription (positive log2ratio); grey represents molecules present in the dataset, but did not meet the defined cut-off for differential transcription; white represent molecules, which were included in the network because of their known relationships with other detected molecules, but they were not present within the data set. (TIF) [file ppat.1003448.s001.tif]

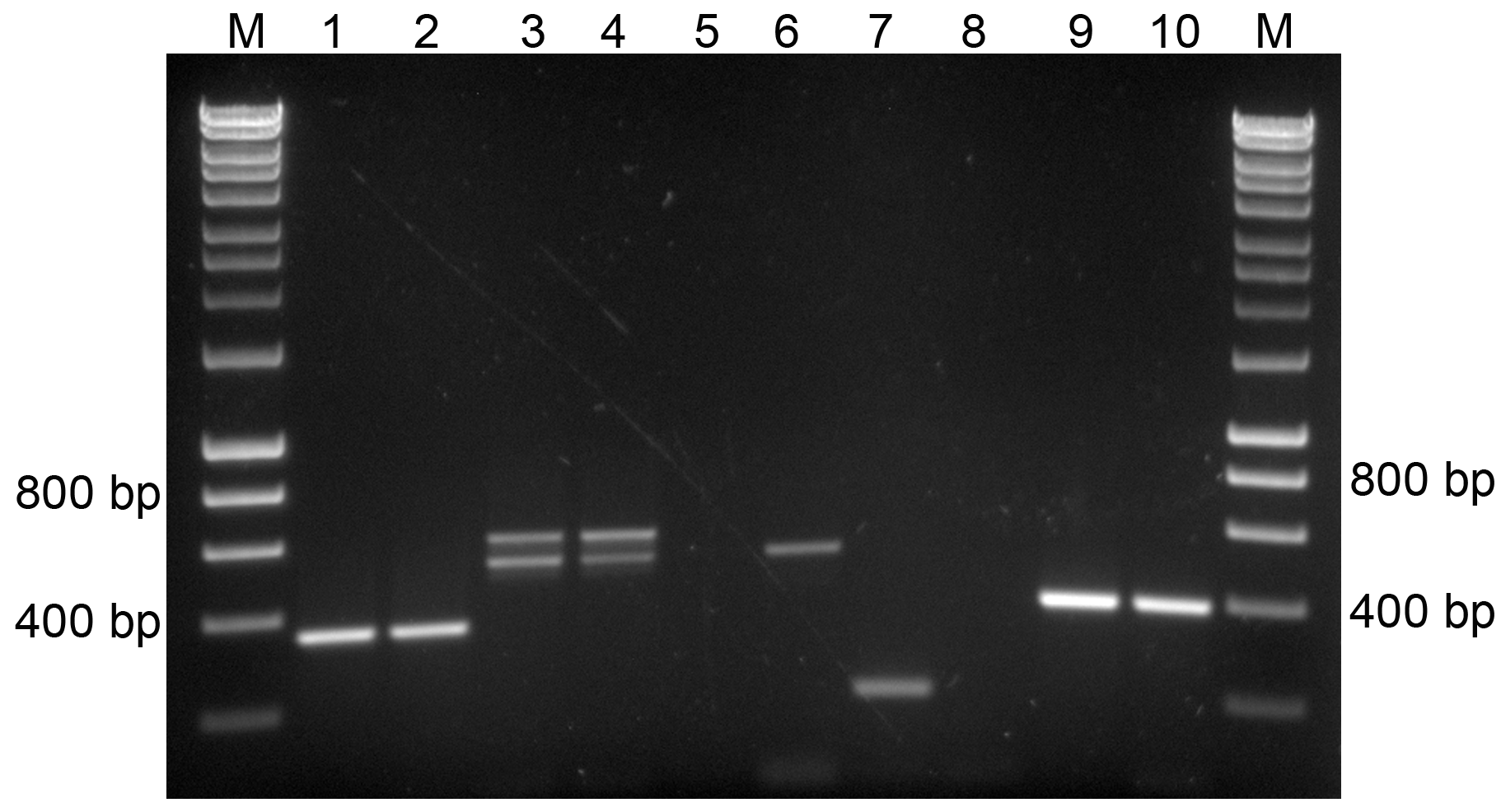

Supplement: Figure S2 — RT-PCR with organ-specific RNA confirmed and complemented in situ -hybridization data. Shown are RT-PCR results with organ-specific RNA of purified testes (lanes 1, 3, 5, 7, 9) and ovaries (lanes 2, 4, 6, 8, 10) indicating the presence of transcripts of hippocalcin (lanes 1, 2), ORAI-1 (lanes 3, 4), the egg-shell precursor gene (lanes 5, 6), and calmodulin-4 (lanes 7, 8). As positive control hsp70 was used (lanes 9, 10), which was shown to be widely expressed [94] serving as a suitable control also for gonad tissue [Hahnel et al., submitted]. The two bands in lanes 3 and 4 resulted from two different splice forms of ORAI-1 (Smp_076650.1, Smp_076650.2; expected products 491 bp and 532 bp), which were detected both by the used primers. M: HyperLadder (Bioline). (TIF) [file ppat.1003448.s002.tif]

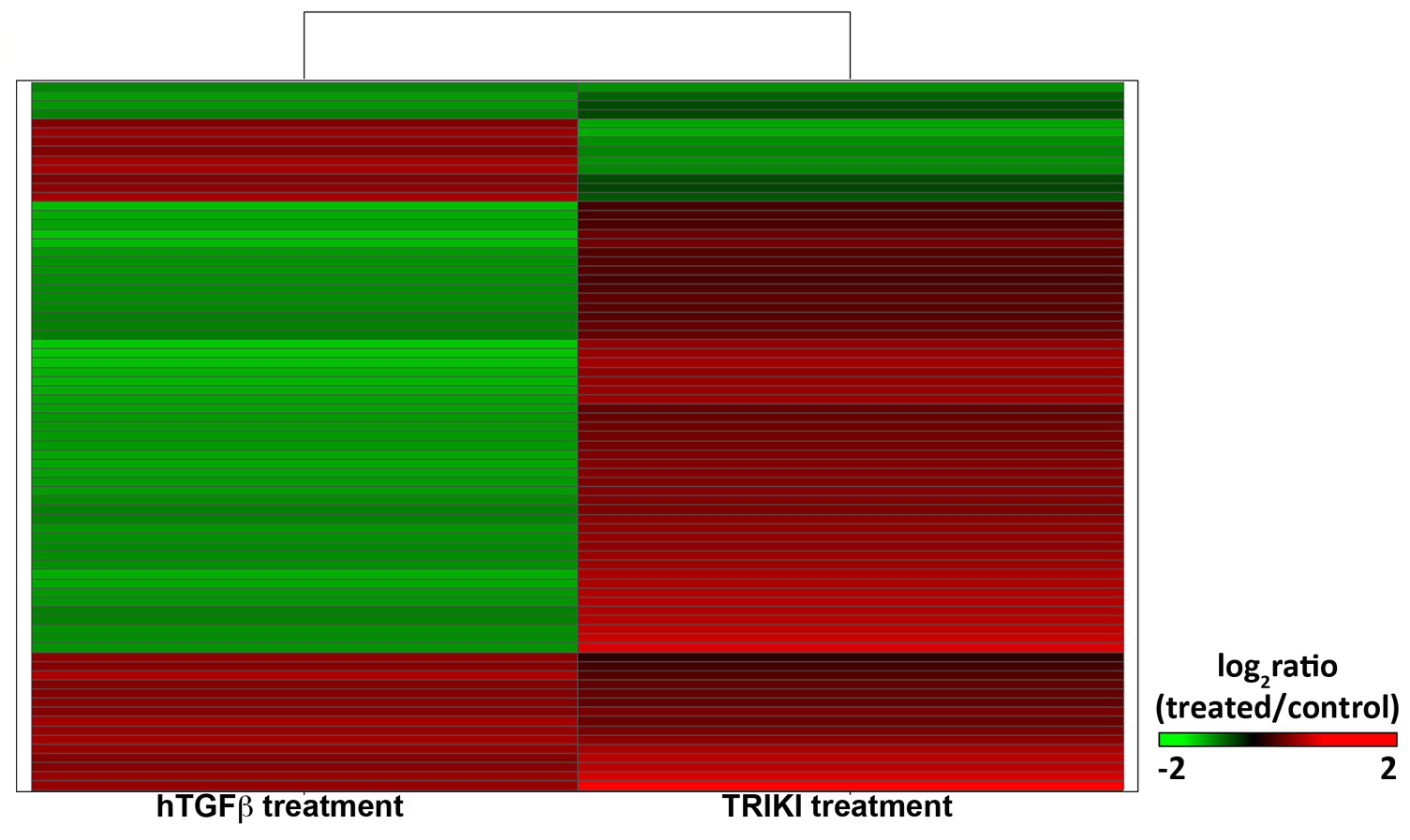

Supplement: Figure S3 — Comparison of differentially transcribed genes following hTGFβ- or TRIKI-treatment. Hierarchical clustering of genes differentially transcribed after either hTGFβ stimulation [27] or TRIKI-induced inhibition of female schistosomes. Each line represents one of 77 genes that were identified to be differentially transcribed in both microarray analyses. The comparison was done using the mean log2ratio (treated/control) of transcription of these genes; genes with an enhanced transcription in treated compared to control are shown in red, and with a repressed transcription in green. (TIF) [file ppat.1003448.s003.tif]
