# Supplementary material for: Transcriptome Analyses of Inhibitor-treated Schistosome Females Provide Evidence for Cooperating Src-kinase and TGFβ Receptor Pathways Controlling Mitosis and Eggshell Formation
Source: PLoS Pathog. 2013 Jun 13;9(6):e1003448. doi: 10.1371/journal.ppat.1003448 (PMC3681755; doi:10.1371/journal.ppat.1003448)
Supplement: Table S1 — List of the numbers of significantly differentially transcribed genes following inhibitor treatments (q≤0.03). This table contains a list of the numbers of significantly differentially transcribed genes following inhibitor treatments (q≤0.03). (DOCX) [file ppat.1003448.s004.docx]

Supplementary table S1: List of the numbers of significantly differentially transcribed genes following inhibitor treatments (q ≤ 3%)

| **Treatment** | **Orientation of probe** | **Total number of significantly differentially transcribed genes** | **Number of significantly differentially transcribed genes according to regulation direction** | **Regulation of transcription** |
| --- | --- | --- | --- | --- |
| TRIKI | Sense | 2330 | 565 | Repressed |
|  |  |  | 1766 | Enhanced |
|  | Antisense | 265 | 198 | Repressed |
|  |  |  | 67 | Enhanced |
| Herbimycin A | Sense | 1021 | 302 | Repressed |
|  |  |  | 719 | Enhanced |
|  | Antisense | 160 | 103 | Repressed |
|  |  |  | 57 | Enhanced |
| TRIKI and Herbimycin A | Sense | 411 | 157 | Repressed |
|  |  |  | 254 | Enhanced |
|  | Antisense | 110 | 40 | Repressed |
|  |  |  | 70 | Enhanced |
