# Supplementary material for: Transcriptome Analyses of Inhibitor-treated Schistosome Females Provide Evidence for Cooperating Src-kinase and TGFβ Receptor Pathways Controlling Mitosis and Eggshell Formation
Source: PLoS Pathog. 2013 Jun 13;9(6):e1003448. doi: 10.1371/journal.ppat.1003448 (PMC3681755; doi:10.1371/journal.ppat.1003448)
Supplement: Table S13 — List of primers used for qPCR. (DOCX) [file ppat.1003448.s016.docx]

**Supplementary Table S13: List of primers used for qPCR**

| **Name of molecule** | **Primer name** | **Primer sequence (5’ 🡪 3’)** | **Tm [°C]** |
| --- | --- | --- | --- |
| Calmodulin-4 | Smp_032990-2-f | ATGAATGTTCCAATAACTCGTG | 60 |
|  | Smp_032990-2-r | CTCTTCACTTCTTGACGATTG | 60 |
| Cathepsin S | Smp_139240_f | CCAGAATACGTTGATTGGAGA | 60 |
|  | Smp_139240_r | TGGAGTAAGAGTTCCTGTCTTGA | 60 |
| Eggshell precursor protein | Smp_000430_f | CCGTAAAGGTGGTGGC | 60 |
|  | Smp_000430_r | TTGAATGTTGAATAGCCTTGC | 60 |
| fs800 | Smp_000270_f | CAGCCGAAAAAGTCAAACA | 60 |
|  | Smp_000270_r | CCCTTTTGCATCGTAAGCT | 60 |
| Hsp70 | Smp_106930-f | GCGTGCACTGACTAAGGAC | 60 |
|  | Smp_106930-r | CTGCTTCCCAGTACCCTTG | 60 |
| Sodium/potassium-pump | Smp_015020-f | GGCTGAAAATGGTTTTTGG | 60 |
|  | Smp_015020-r | AGGCAGTTTGACATGTGG | 60 |
| Smp14 | p14-f | CCTATGGCGGTGATTATGG | 60 |
|  | p14-r | GGCTGGGTTTGTAAGTGC | 60 |
| Smp48 | p48-f | GACAAGCATGGTCATGGA | 60 |
|  | p48-r | ATGCTTATCGTGGTCTTTACG | 60 |
| SmSmad 4 | Smp_033950-f | CCTTCTGGGTCCATACTCC | 60 |
|  | Smp_033950-r | CCGTGTAACCGTCAACAGTG | 60 |
| SmTβRI | TGFβRI-f | ATGATCTCACGTGTGCGA | 60 |
|  | TGFβRI-r | GATCCGAAGGGCTGTCTT | 60 |
| SmActRIIb | TGFβRII-f | GGCAGAAAATTCACCCA | 60 |
|  | TGFβRII-r | GGGGCCTGATATCGTAAAG | 60 |
| Snurp | Smp_069880-f | CGGTGGAGATATCAATGC | 60 |
|  | Smp_069880-r | GCAGCACGTCTAGCCTC | 60 |
| Tetraspanin 1 | Smp_155310.1-f | TGTGGATTATTGAATGGATCTG | 60 |
|  | Smp_155310.1-r | GGACATGTTGGATCGAGAA | 60 |
| Tetraspanin 18 | Smp_174190-f | CAAATCGTACCGCTTGTC | 60 |
|  | Smp_174190-r | GCCATCATTAATACGGCC | 60 |
| Tetraspanin-1 | Smp_011560-f | CAAAGAATGCTGGGAACG | 60 |
|  | Smp_011560-r | ACCGCGAATGTAAACGTAA | 60 |
| SmTYR1 | Smp_052070-f | CAGGGACAGCAAGCAATG | 60 |
|  | Smp_052070-r | CTTGAATGTCCAGGACGAA | 60 |
